# Make “Incongruent” to Be “Excellent”: Fluid Compensation in Extremely Incongruent New Products

**DOI:** 10.3389/fpsyg.2022.878039

**Published:** 2022-04-18

**Authors:** Xionghui Leng, You Chen, Xinyu Song, Xiaoyu Zhou, Xi Li

**Affiliations:** ^1^School of Economics and Management, East China Jiaotong University, Nanchang, China; ^2^College of Foreign Languages, Shenzhen University, Shenzhen, China

**Keywords:** extremely incongruent new products, meaning maintenance model, fluid compensation, anxiety, self-esteem

## Abstract

Under fierce market competition, firms are accelerating the pace of product innovation, which may bring extremely incongruent new products. Generally, consumers are unfamiliar with extreme incongruence, resulting in passive product evaluation and a low success rate. This study draws on the meaning maintenance model of fluid compensation theory to examine how extremely incongruent new products influence product evaluation. Three experiments indicate that consumers’ product evaluation of extremely incongruent new products is significantly lower than that of congruent products, and it can be notably improved by three ways of fluid compensation. The findings revealed that anxiety moderates the relationship between extremely incongruent new products and product evaluations, and further self-esteem moderates the relationship on the main effects. Overall, our research reconciles divergent findings on the effect of extremely incongruent new products on the effects of product evaluation and further provides rich managerial implications for product innovation management.

## Introduction

With the progress of technology and fierce market competition, homogenous products are hard to meet consumers’ diverse needs, who gradually shift their pursuit from material utility to connotation of products and pay more attention to unconventional products. A variety of approaches have been conducted on the practicality and aesthetic perception and identification of products to enable the consumers to easily identify their desired product from similar ones (de Guimarães et al., 2021; Duong et al., 2022). For instance, Dyson Bladeless Fan, LG Aluminum Box Hidden Display TV, and Astrodel Multi-function Heating/Air Purifier are innovative products. Product innovation has become a hot topic in academic research in the past 20 years (Najafi-Tavani et al., 2018; Xie et al., 2019; Awan et al., 2021). However, some innovative products are extremely incongruent with the extant schema of product categories, leading to low acceptance and negative evaluation and even failure of the new products (Noseworthy and Trudel, 2011; Jhang et al., 2012), such as crystal colorless Pepsi, and Pamia smokeless cigarette. Product incongruity refers to the mismatch between a product and other varieties of the same category (Noseworthy and Trudel, 2011; Jhang et al., 2012; Taylor and Noseworthy, 2020). Statistics also show that the failure rate of product innovation is very high in both traditional and emerging industries (Gourville, 2006), with failure estimates ranging from 40 to 90%. As a result of social development and technological progress, enterprises must carry out product innovation to occupy market share, and it is inevitable to introduce new products. How to solve the problem of low product evaluation caused by extremely incongruent products has become the focus of research on innovative products.

Previous studies on incongruent new products mainly adapted the schema congruity theory (Lee et al., 2020; Wang et al., 2020; Zhou et al., 2021), which argues that once individuals can deal with incongruent products based on their knowledge of existing products and their evaluation of the products can be improved, namely assimilation effect (Meyers-Levy and Tybout, 1989). When the level of incongruity exceeds a threshold value (extreme incongruity), the capacity of handling the product will decrease exponentially, and so will the evaluation (Noseworthy et al., 2011). At present, there are many studies on consumers’ evaluation and adoption mechanism of moderately incongruent new products, but only a few scholars have paid attention to extremely incongruent products.

Although moderately incongruent products have higher acceptance, extremely incongruent products are also meaningful to enterprises (Jhang et al., 2012; Huang and Wan, 2019; Zhu et al., 2020). From the subjective perspective, due to the serious homogenization of products, enterprises will carry out extremely incongruent product design in order to better distinguish their products from similar products (Noseworthy and Trudel, 2011; Taylor and Noseworthy, 2020), which will lead to better brand awareness (innovative brand; Clemente et al., 2013; Huang and Wan, 2019). From the objective point of view, the invention of new technology will inevitably make product design extremely incongruent with the original product schema. Of course, it is undeniable that moderate innovation on the basis of original products may make them more acceptable to customers (Noseworthy and Goode, 2011; Bagga et al., 2016), and it is a better choice if consumers’ fear and rejection can be reduced at a constant rate of product replacement.

Although existing studies attempt to improve the evaluation of extremely incongruent new products from the perspectives of tactile transmission (Liu et al., 2018), changing product display (Li et al., 2018a; Li et al., 2021; Zhou et al., 2021), and improving cognitive flexibility (Jhang et al., 2012), these approaches are applicable to limited products and channels. For example, touch is more suitable for products with rich tactile attributes (such as computer mice), not able to be extended to all visually incongruent products and products with incongruent functions. It is of weak applicability to the current omnichannel retail trend. The meaning maintenance model in psychology argues that when meaning is violated, a series of behavioral motivations will be triggered in a bid to maintain the meaning (Proulx and Heine, 2010). The extreme incongruity between the product and the existing schema damages the meaning of the product, and the meaning can be compensated through meaning maintenance strategies. The fluid compensation strategy also indicates that when meaning is unable to be obtained from the field, it can be maintained by affirming other values or beliefs (Forehand et al., 2021; Mandel et al., 2021; Wang et al., 2022). Based on the meaning maintenance model in the field of psychology, this study attempts to apply fluid compensation theory to improve the evaluation of extremely incongruent products and provide solutions for more types of incongruent products or sales channels in enterprises.

**Figure 1:**
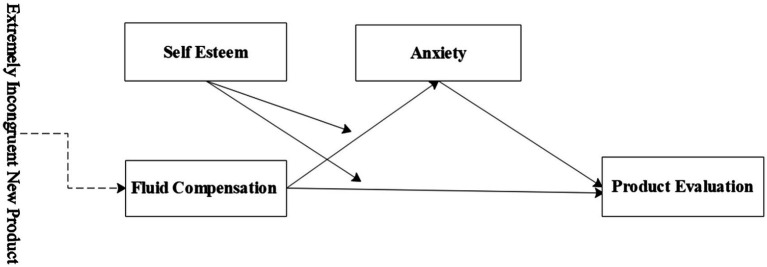
Research framework.

In this study, theories such as extremely incongruent product, meaning maintenance model, and fluid compensation are first expounded; then, theoretical inference and hypotheses are made that the meaning violation of extremely incongruent products may arouse anxiety, and fluid compensation for incongruent products can reduce the level of anxiety aroused and improve product evaluation, and three experiments were conducted to verify the theoretical hypotheses. Experiment 1 mainly validates the effect of fluid compensation on the evaluation of the extremely incongruent product. Experiment 2 further explores the mediating mechanism of the fluid compensation effect. Experiment 3 verifies the boundary conditions of fluid compensation based on the first two experiments. This study aims to provide new ideas for enterprises to promote extremely incongruent new products through a series of studies. The research framework is shown in Figure 1.

## Literature Review and Hypotheses Development

### Extremely Incongruent New Products and Fluid Compensation

The schema congruity theory initially proposed a dynamic non-monotonic relationship between product incongruity and product evaluation (Meyers-Levy and Tybout, 1989; Lee et al., 2020). Consumers have schematic cognition of a product type with moderate incongruity; the incongruity can be solved with the minimum cognitive efforts of consumers to make the product pleasing and gain favorable evaluation (Meyers-Levy and Tybout, 1989; Lee et al., 2020). However, extreme incongruent new products bring high psychological novelty, which is unable to be solved through the existing schema and induce consumers unwilling to accept extremely incongruent products resulting in lower evaluation (Alexander et al., 2008; Pleyers, 2021).

Fluid compensation originated from the meaning maintenance model (Proulx and Heine, 2010). This model refers to the reaction and behavioral process triggered by the meaning violation brought about by the destruction of the original understanding that is caused by the contradiction between individuals’ expectancy based on their own understanding and their experiences (Proulx and Inzlicht, 2012). Such meaning violation can be triggered by uncertainty, dissonance, and violation of expectancy (Major and Townsend, 2012). Uncertainty refers to the inability of an individual to make accurate predictions about the surrounding environment; dissonance refers to the subordination of different logical rules between an individual’s idea and behavior; and violation of expectancy refers to the unconformity between the facts and the individual’s self-image-based expectancy, worldview-based expectancy, or category-based expectancy. Extreme incongruent new products belong to high-level meaning violations brought by incongruity. Meaning violation arouses aversive emotions to stimulate the motivation to maintain the meaning, which allows individuals to change their existing behavioral patterns to alleviate the aversive sentiment and compensates for the missing sense of meaning (Proulx and Inzlicht, 2012).

Fluid compensation refers to compensation made with the assistance of beliefs outside the field when it is unable to be made through the beliefs within the field after the meaning is violated (Tesser, 2000). For example, once the participants encounter obstacles in reading surreal short stories, they tend to identify more with their culture (Proulx and Inzlicht, 2012), and it is a pattern that unconsciously enhances connectivity in unrelated domains (Taylor and Noseworthy, 2020). The extant means of fluid compensation mainly include semantic associations (i.e., dominant brand), ethical beliefs (i.e., green consumption), affirming personal culture (i.e., ethnocentric theory), etc. Since fluid compensation occurs at the unconscious level, consumers may strengthen semantic associations after experiencing a meaning violation of expectancy (Randles et al., 2011). The dominant brand is one of the strongest semantic associations to a given product category (i.e., Nike’s dominance in the sports brands). Dominant brands are considered typical and representative of this category, and they are recognized earlier than non-dominant brands (Zhou et al., 2021). Accordingly, when consumers are faced with extremely incongruent products, the lack of such meaning will be partly made up by affirming the dominant brand. In addition, meaning can be compensated by means of affirming ethical beliefs, such as a preference for environment-friendly (i.e., green) products (Waheed et al., 2020; Awan et al., 2021; Jabeen et al., 2021; Stojčić, 2021; Kolling et al., 2022), assuming that buying these products is conducive to improve society and ease tensions arising from the violation of expectancies (Qiu et al., 2020; Sadiq et al., 2021). Besides, people will affirm their cultural identity through ethnocentric consumption after experiencing a violation of expectancies (Narang, 2016; Li and Katsumata, 2020). Typical examples are studies on the country-of-origin effect, and people prefer to pay for products from the region they identify with.

The schema congruity effect builds on the concept that people actively seek meaning (Bruner, 1990). Research on new products has found that consumers may generate positive feedback on a new product only when they do not need to abandon or restructure their existing meaningful understanding of the product type (Campbell and Goodstein, 2001; Noseworthy et al., 2011; Noseworthy and Trudel, 2011). The meaning of extremely incongruent products is unable to be resolved in the established product belief systems and thus violated, which often brings adverse evaluation and relies on aversive motivation (Proulx and Inzlicht, 2012; Noseworthy et al., 2014), and individuals may regain a sense of meaning through fluid compensation to affirm a commitment to another framework of meaning (Wang et al., 2020). The motivation for people to compensate for unrelated fields is not to address incongruity, but to mitigate aversion due to meaning violation through an unconscious way (Major and Townsend, 2012). Overall, consumers may raise their evaluation of extremely incongruent new products by affirming belief systems in irrelevant fields. Therefore, the first hypothesis was proposed:

*Hypothesis 1*: Fluid compensation influences the evaluation of extremely incongruent products.

### Mediating Effect of Anxiety

The literature on schema congruity and meaning maintenance can be unified under a common emotional mechanism, that is, anxiety aroused by dissonance. Related studies suggest that anxiety is a vague and unpleasant mood that comes with the premonition that something uncertain is about to happen (Ali et al., 2021; Simpson et al., 2021). The information theory of emotion suggests that people tend to classify their brief empirical emotions into judgments if they are asked to evaluate something, so anxiety has an impact on information processing (Hong et al., 2019) and cognitive emotion (Kamalou et al., 2019), and it also triggers preferences for safer options to provide a sense of control to reduce risk and uncertainty (Gruda and Hasan, 2019; Brailovskaia and Margraf, 2021). The anxiety aroused by uncertainty is mainly due to the extreme difference in the appearance of extremely incongruent products, which leads to the violation of the meaning model related to the original product schema (Proulx and Inzlicht, 2012). Therefore, the meaning of extremely incongruent products is uncertain. To some extent, the difference in appearance may also be due to changes in product functions (such as extremely incongruent stress relief mouse), and new functions bring uncertainty in product experience. Anxiety can be divided into trait anxiety and state anxiety. The former is a learned behavioral tendency that reflects significant individual differences in the frequency and intensity of human responses to tension, while the latter is a temporary emotional state, a response state that only occurs in a specific situation.

Existing studies on schema congruity demonstrate that since extremely incongruent products challenge the extant schematic cognition of products and bring about uncertainty, people’s autonomic neurophysiological response is triggered, manifesting as anxiety (Noseworthy et al., 2014; Holte and Ferraro, 2020). When the source of such an anxiety state is unclear, the impact on decisions will not be limited to local-range adjustments (Raghunathan et al., 2006; Wang et al., 2022). Therefore, for the unconscious anxiety state awakened by extremely incongruent new products, we can expand the scope of adjustment, for instance, using fluid compensation, to strengthen the affirmation of the belief system in unrelated fields to compensate for the meaning violation caused by extreme incongruity to relieve anxiety (Taylor and Noseworthy, 2020). This is consistent with findings in the literature on meaning maintenance that support anxiety awakened by expectancy violation and encourage consumers to compensate by means of affirming unrelated belief systems (Proulx and Heine, 2008) to reduce the degree of autonomous responses (such as anxiety aroused by extreme incongruity) and improve the evaluation (Noseworthy et al., 2014). In conclusion, the following hypothesis is proposed:

*Hypothesis 2*: The effect of fluid compensation for extremely incongruent products on product evaluation is mediated by the level of anxiety.

### Moderating Effect of Self-Esteem

Self-esteem is closely related to the pattern of meaning identification in individuals’ working memory (Sowislo and Orth, 2013; Li et al., 2018b; Dhandra, 2020) and indicates their expectation of success, degree of confidence in failure (Dumas et al., 2020; Xie et al., 2020) and a good sense of themselves (Sun et al., 2017; Tibber et al., 2020). Accordingly, self-esteem impacts significantly on an individual’s cognitive system and behavioral control system and affects the processing of incoming information through experience-based cognitive representations (generally oneself).

Many studies have found a close relationship between self-esteem and anxiety (Kim and Koh, 2018), for instance, the Anxiety Buffer Hypothesis and the Mortality Salience Hypothesis. In addition, Zohreh et al. (2007) pointed out that full self-concept and propensity toward high-level self-esteem not only protect state anxiety but also suppress trait anxiety. As individuals with high-level self-esteem have strong self-efficacy, the adjustment function of self-esteem is effectively performed to enable the individuals to act more actively and positively under situations prone to anxiety and have a more rational and peaceful attitude so that they experience less anxiety (Berger et al., 2017).

People with low-level self-esteem are more likely to develop anxiety than those with higher-level self-esteem (Sowislo and Orth, 2013), and they are more likely to stimulate self-protection mechanisms and maintain meaning through compensatory measures. Specifically, people with low-level self-esteem tend to perceive that they are unable to solve the threats posed by extremely incongruent products and thus enhance anxiety and their psychological defensive behaviors induce them to accept the meaning maintenance of fluid compensation. However, people with high-level self-esteem have a lower perception of extreme incongruity than those with low-level self-esteem, and they are more confident that they can solve the meaning violation caused by this incongruity through their personal efforts, not need to use other means, such as fluid compensation to maintain the meaning so that the motivation for meaning maintenance is weakened. Besides, people with high-level self-esteem tend to have more positive self-evaluation by recognizing their self-worth, facilitating them to face the meaning threats caused by the incongruence in the product schema incongruity, and within the cognitive framework, the physiological arousal characteristics of anxiety can be reduced by changing the thoughts that promote anxiety (Pyszczynski et al., 2004), and therefore, the positive evaluation of people with high-level self-esteem benefit them to reduce the level of anxiety aroused by extreme incongruity, and they are not likely to trigger their psychological defensive mechanisms. Accordingly, the following hypothesis is proposed:

*Hypothesis 3*: The effect of fluid compensation for extremely incongruent products on product evaluation is moderated by the level of self-esteem.

## Methodology

This study conducted three sub-studies. In study 1, we conducted Experiment 1 to examine the effects of fluid compensation on the evaluation of extremely incongruent products. In study 2, we conducted Experiment 2 to examine the mediating role of anxiety on the relationship between fluid compensation and the evaluation of extremely incongruent products. In study 3, we conducted Experiment 3 to examine the moderating effect of self-esteem on the relationship between fluid compensation for the evaluation of extremely incongruent products. The approaches of fluid compensation adopted in the three experiments were a dominant brand (Study 1), green consumption (Study 2), and ethnocentric consumption (Study 3), respectively. We selected three kinds of stimuli, including tissue paper (Study 1), vacuum cup (Study 2), and computer mouse (Study 3). First, the three types of products were selected for their high familiarity and strong perceptual prototype, which could arouse the necessary product schema of the individual; second, these three types of products not only took into account the requirements of extremely inconsistent visual products, but also of extremely inconsistent products brought by technological innovation (extremely incongruent stress relief mouse); third, these three types of products had also been used by prior work to explore the impact of product congruity on product evaluation (Li et al., 2018b; Taylor and Noseworthy, 2020).

### Study 1

The first study examined whether fluid compensation impacts the evaluation of extremely incongruent products. The pretest procedure followed the experimental procedure on new products of Noseworthy et al. (2014). First, three kinds of tissue paper with very different appearances were selected, and 163 participants in a comprehensive university in south China were chosen and randomly divided into three groups. Participants of each group viewed a picture of one of the tissues on a computer, and they were asked to rate the appearance congruity of the products. Measurement scales of congruity applied the items developed by Noseworthy and Trudel (2011) with the seven-point scale, and the example items were “Compared with common products, I think the appearance typicality of this product (reverse coding) /commonness complies with your expectancy” (*α* = 0.873). All participants in the experiment will receive a certain amount of cash reward. The results showed that the incongruity of the three types of tissue paper was significant [*M_1_* = 5.46, *M_2_ =* 4.59, *M_3_* = 2.24, *F* (1, 161) = 61.97, *p* < 0.001, *η*^2^ = 0.588], referring to the dividing criteria of Noseworthy et al. (2011) and Jhang et al. (2012). In this study, the black tissue paper was taken as the extremely incongruent product and the white tissue paper as the congruent product for the experiment (Table 1).

**Table 1:** Pretest of tissue paper.

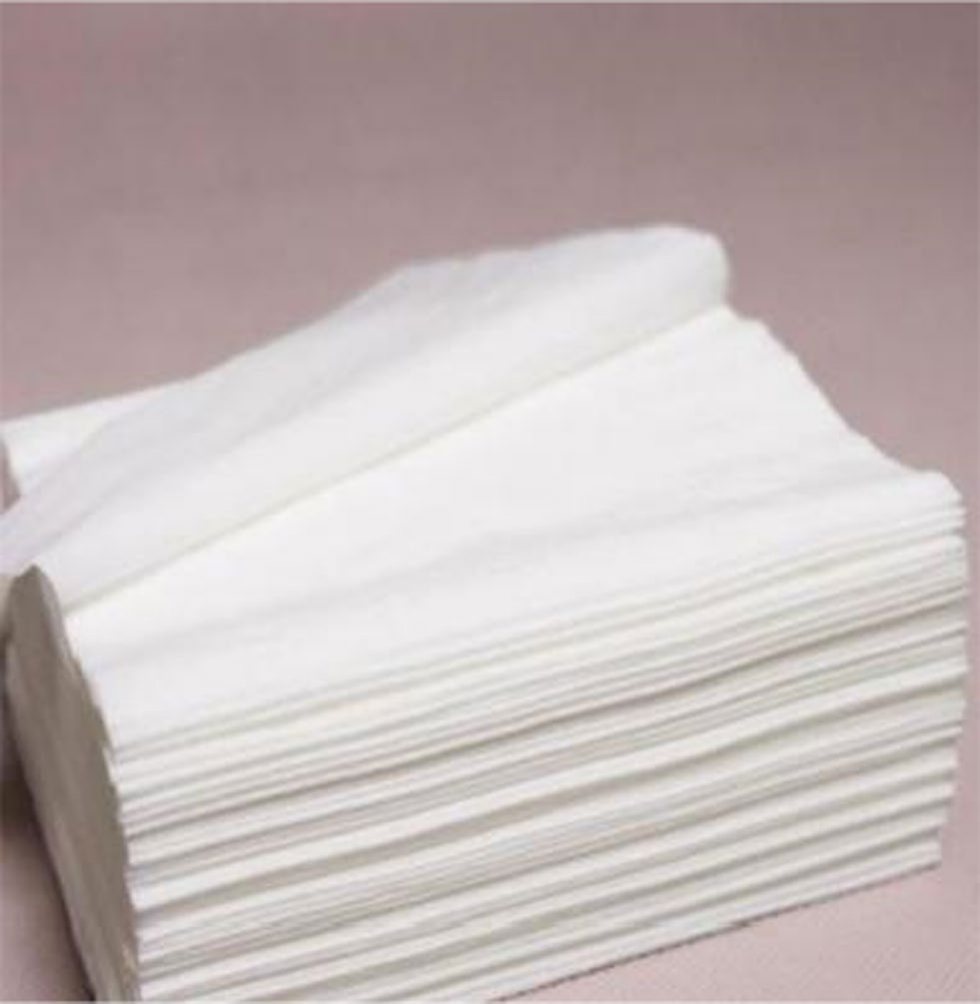 Congruent Product	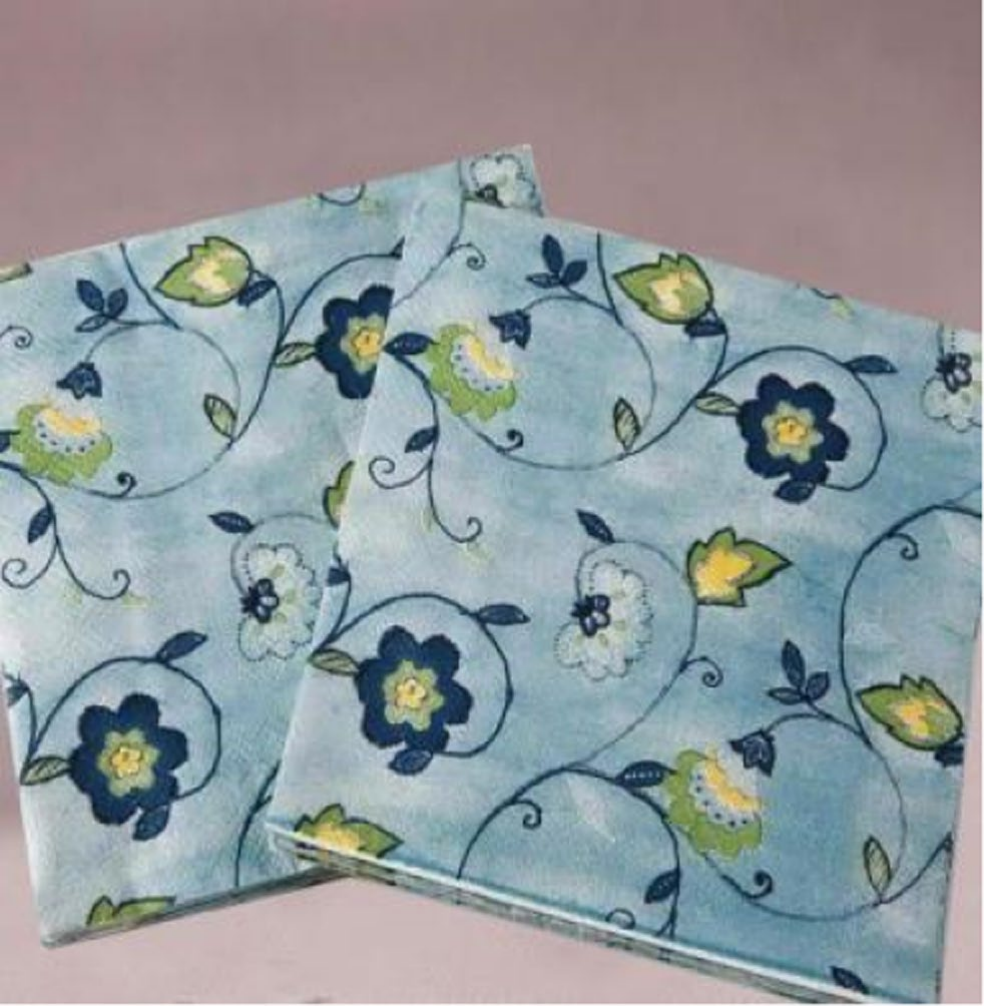 Moderately Incongruent Product	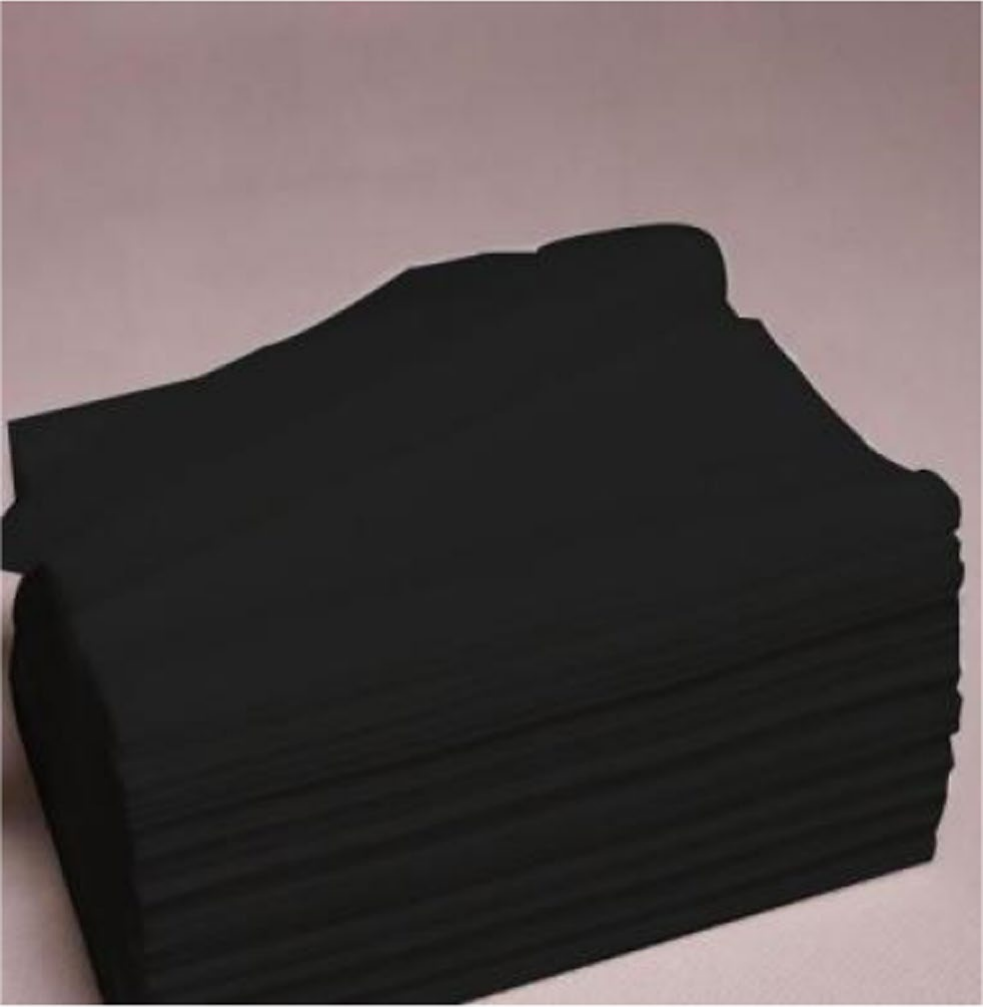 Extremely Incongruent Product
M1 = 5.46	M2 = 4.59	M3 = 2.24
SD = 1.11	SD = 1.04	SD = 1.30

The brand description for each kind of tissue paper was designed with the same number of words. The dominant brand was described as “the brand of this tissue paper is very typical and representative, closely correlated with the product and almost known by everyone,” and the non-dominant brand was described as “the brand of this tissue paper is extremely common and niche, weakly correlated with the product and only known by a few people.” The product presentation consisted of a product picture (picture of the extremely incongruent product), product name, and brand description; the pictures of the products processed by Photoshop software are of the same size and put above the brand description.

In this experiment, 128 participants from a comprehensive university in south China were randomly divided into two groups: dominant brand group and non-dominant brand group, and they were asked to choose which brand the tissue paper was more likely to be based on the description, *Mind Act Upon Mind* or *Meeg*. Before differences in brand perception were examined, the above answers were coded, namely *Mind Act Upon Mind* was coded as 1, *Meeg* as 0, *α* = 0.73. It turned out that in the description of dominant brand, more participants chose *Mind Act Upon Mind* (70% vs. 30%), whereas, in the description of non-dominant brand, more participants chose *Meeg* (80% vs. 20%), *Pearson χ*^2^ (1) = 32.28, *p* < 0. 001, showing that the operations were effective and can be used in the experiment. In addition, participants in the pretest would not participate in the subsequent formal experiment.

#### Experimental Design and Procedures

In study 1, we designed a 2 (product congruity: congruent product vs. extremely incongruent product) × 2 [fluid compensation: with (dominant brand) vs. without (non-dominant brand)] between-subjects factorial design. G*Power 3.1.9.2 (Faul et al., 2007) was used to estimate the planned sample size. Setting a significance level of α = 0.05, estimated power of 1-β = 0.80 (requiring more than 0.8, Faul et al., 2007; Taylor and Noseworthy, 2020), and medium effect size *f* = 0.25 (small: *f* = 0.1, medium: *f* = 0.25, large: *f* = 0.4) as the standards to calculate the minimum sample size of the effects (main effects and interaction effects) of all factors. This setting refers to previous similar experiments (Huang et al., 2021; Miao et al., 2021), and the result was 128. Considering the possibility of participants not responding seriously in the experiment and the requirement of a greater estimated power, more participants were selected.

In this experiment, we randomly selected 200 participants from a comprehensive university in south China, and they were randomly assigned to four groups and completed the study on the computer in the laboratory. All participants were told that they were taking part in a consumer survey to explore consumers’ opinions about a new tissue paper product, and the true purpose of the experiment was concealed from the participants. Then, white or black tissue paper products were displayed on the computer screen for the participants to evoke the necessary patterns. At the bottom of the product picture, the two groups of participants would see product descriptions of the dominant brand and non-dominant brand, respectively. A minute later, the participants were directed to the next page to complete the measurement items.

Additionally, all participants were asked to answer nine random items on a scale of seven points. Specifically, four items are measured for overall evaluation (*α* = 0.851; Campbell and Goodstein, 2001), three items for perceived typicality (*α* = 0.767; Noseworthy and Trudel, 2011), two items for incongruity resolution (*α* = 0.745; Jhang et al., 2012). Then, they were asked to fill in the demographic information. The detailed information is shown in Table 2.

**Table 2:** Scale items of different variables.

Variables	Items
Product Evaluation	1. I think this product is very unfavorable/ very favorable.
	2. I think this product is bad/ good.
	3. I think this product is unappealing/ appealing.
	4. I think this product is undesirable/ desirable.
Perceived Typicality	1. Compared to the general products, I think this product looks not at all novel/ extremely novel (r).
	2. Compared to the general products, I think this product looks very unlikely/ very likely.
	3. Compared to the general products, I think this product matches my expectations not at all well/ very well.
Incongruity Resolution	1. The extent of the new product made sense to me (“makes no sense/ makes sense”).
	2. I understand the rationale of the product (“disagree/ agree”).

*“R” means the reverse scoring item*.

#### Results and Discussion

##### Operation Verification: Perceived Typicality of Products

In line with reverse items, invalid questionnaires that based on the same attitude except neutral in the positive and negative items were eliminated, and 170 valid participants were left. The effective rate was 85%, which still met the sample requirements. Among them, 43 were in the incongruent non-dominant brand product group, 42 were in the incongruent dominant brand product group, 42 were in the incongruent non-dominant brand product group, and 43 were in the incongruent dominant brand product group. Among them, 57.6% were female, 78.8% were between 18 and 25 years old, 95.3% had a bachelor’s degree or above, and 87.1% had an income of less than 3,000 yuan per month. The result of data analysis showed that in terms of products’ perceived typicality, the extremely incongruent products were significantly lower than the congruent products [*M_extremely incongruent_* = 3.71, *SD* = 1.04, *M_congruent_* = 4.02, *SD* = 0.71, *F* (1, 168) = 5.44, *p* = 0.021, *η*^2^ = 0.128], indicating once again that the operation was valid. In addition, the main effect of fluid compensation is not significant [*F* (1, 168) = 2.17, *p* = 0.142, *η*^2^ = 0.042], indicating that fluid compensation may not have changed the perceived typicality to eliminate its influence.

In terms of product evaluation, the main effect of product congruity was significant, and the extremely incongruent product was significantly lower than the congruent product [*F* (1, 166) = 6.22, *p* = 0.014, *η*^2^ = 0.144], and the main effect of fluid compensation was also significant, the evaluation of dominant brand was significantly higher than that of the non-dominant brand [*F* (1, 166) = 10.24, *p* = 0.002, *η*^2^ = 0.232], and the interactive effect was also significant [*F* (1, 166) = 5.90, *p* = 0.016, *η*^2^ = 0.136]; further simple effect analysis showed that for extremely incongruent products, the product evaluation of dominant brand was significantly higher than that of the non-dominant brand [*F* (1, 166) = 15.84, *p* < 0.001, *η^2^* = 0.348], but for congruent products, there was no significant difference between the two brands [*F* (1, 166) = 0.298, *p* = 0.586, *η*^2^ = 0.008]. In terms of incongruity resolution, the main effect of product congruity was significant [*F* (1, 166) = 8.90, *p* = 0.003, *η*^2^ = 0.204], but the main effect of fluid compensation was not significant [*F* (1, 166) = 0.514, *p* = 0.475, *η^2^* = 0.012], demonstrating that fluid compensation may not bring about incongruity resolution and excluding the possibility of incongruity resolution as a mediating variable. Moreover, demographic variables such as gender, age, education, and income had no significant effect on product evaluation. The specific results are shown in Table 3.

**Table 3:** Differences between variables for different products.

	Congruent New Product		Extremely Incongruent New Product	
	Non-dominant brand (without fluid compensation)	Dominant brand (with fluid compensation)	Non-dominant brand (without fluid compensation)	Dominant brand (with fluid compensation)
Product Evaluation	4.29 (0.88)	4.40 (0.80)	3.60 (1.17)	4.39 (0.74)
Perceived Typicality	4.05 (0.78)	4.00 (0.64)	3.48 (1.04)	3.93 (1.00)
Incongruity Resolution	4.49 (1.16)	4.34 (1.09)	3.64 (1.51)	4.05 (1.12)
Cell Sizes	43	42	42	43

In study 1, we conducted experiment 1 to test Hypothesis 1 that fluid compensation influences the evaluation of extremely incongruent products. The result supports this hypothesis. Consumers’ evaluation of extremely incongruent products was lower than that of congruent products. By means of fluid compensation, the meaning violation brought about by the extremely incongruent products could be compensated to improve the product evaluation. As for congruent products, since the meaning was not violated, fluid compensation did not have a significant effect on product evaluation nor bring about incongruity resolution. This in turn proves that fluid compensation does improve product evaluation by compensating for incongruity with meaningful content outside the domain, but the product incongruity is not solved yet, and the meaning of the product is compensated in other ways. Experiment 2 would be designed to further explore the mediating mechanism of the effect of fluid compensation on the evaluation of extremely incongruent products.

### Study 2

A pretest was designed to examine the extremely incongruent products in study 2, and the product category chosen for the congruity manipulation was vacuum cup. A total of 163 college students that were used in Study 1 pretest were randomly divided into three groups to conduct a congruity test on three vacuum cups with an obviously different appearance. The procedure of Experiment 2 follows Experiment 1. Due to the follow-up experiment only studying the extremely incongruent products, in line with the data results, the vacuum cup (cactus vacuum cup) with appearance typicality lower than 3 which is the lowest [*M_3_* = 2.50, *F* (1, 161) = 23.65, *p* < 0.001, *η*^2^ = 0.489] would be used in the experiment as the visually extremely incongruent new product (Table 4).

**Table 4:** Pretest of vacuum cups.

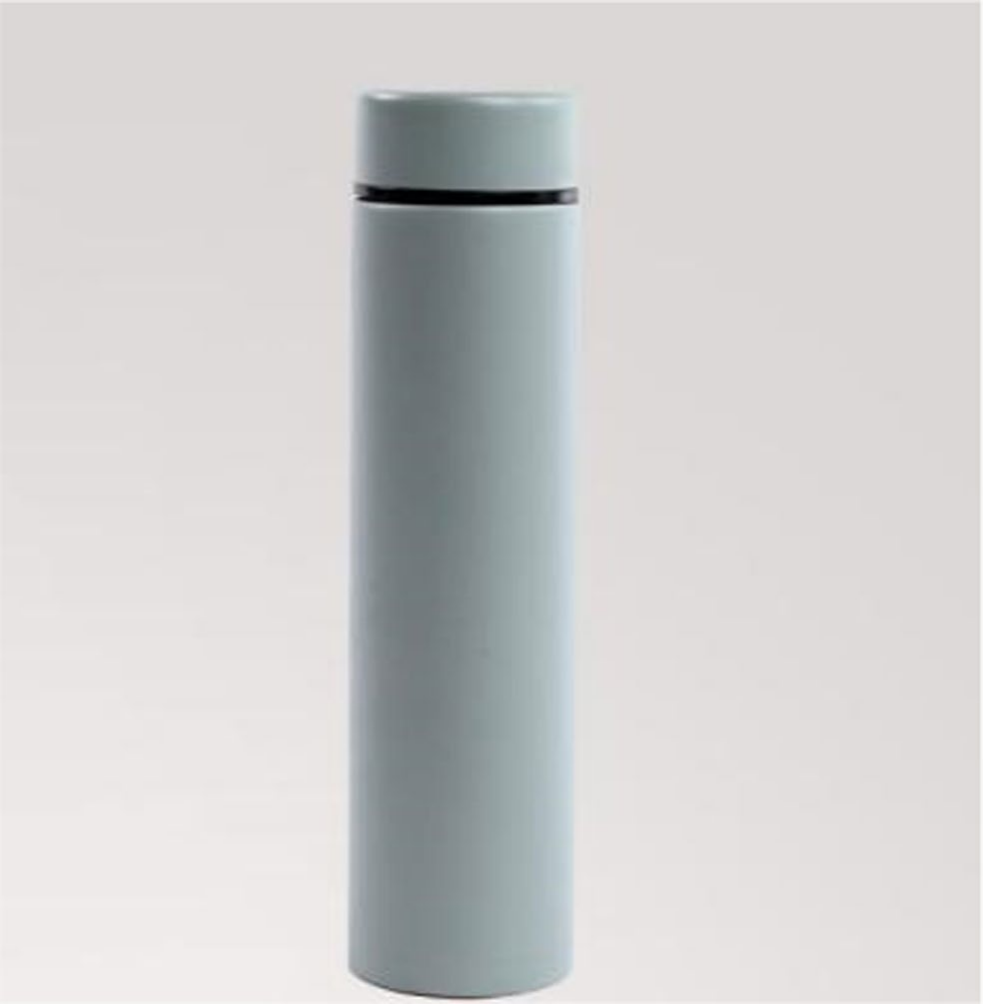 Congruent Product	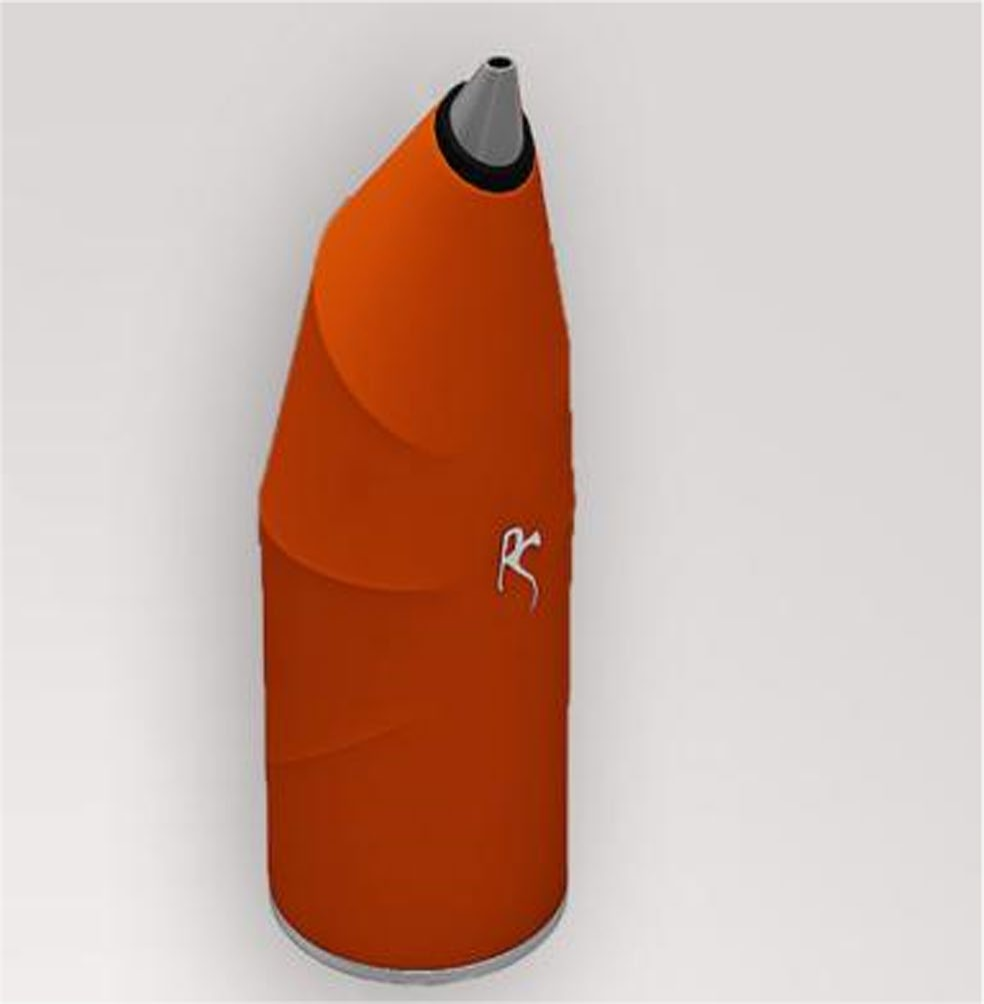 Moderately Incongruent Product	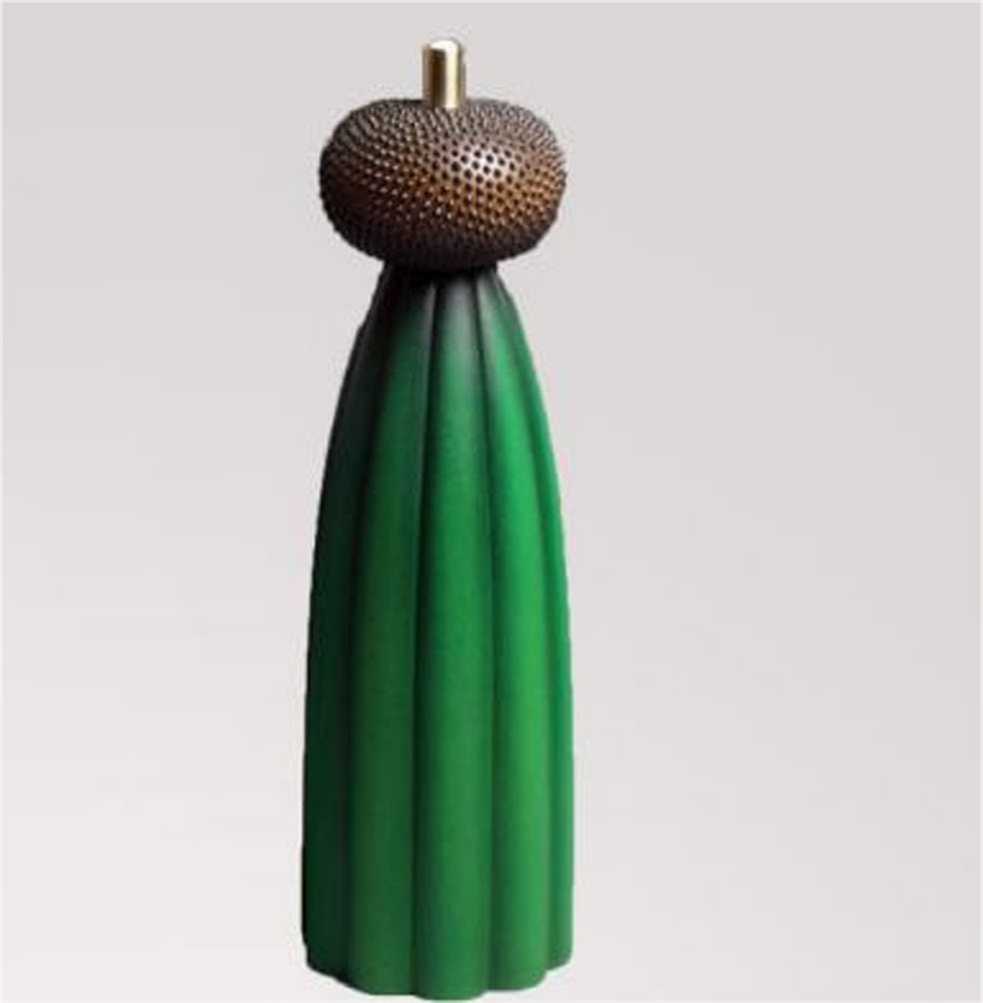 Extremely Incongruent Product
M1 = 4.98	M2 = 3.28	M3 = 2.50
SD = 1.12	SD = 1.01	SD = 1.10

Green consumption consumer behavior was applied to estimate the fluid compensation. Product descriptions with the same number of words were designed for vacuum cups. In particular, the description of a green product was that “This vacuum cup uses green and environment-friendly high-quality raw materials which can be reused, and the production process is of low-carbon emission,” and the non-green product was described as “This vacuum cup adopts double vacuum technology, superb thermal insulation, seven layers of exquisite technology, and 360-degree Celsius leakproof sealing,” and the remains were in line with the process of Experiment 1.

The remaining 128 participants in the pretest of Study 1 were randomly divided into two groups. Since the study showed that the green product was pro-social, a seven-point scale was applied: people who buy this product are (a) friendly, (b) caring, and (c) altruistic (*α* = 0.784; Griskevicius et al., 2010), and the results showed that the prosocial perception of green product was significantly higher than that of non-green product [*M_green product_* = 5.13, *M*_non-green product_ = 4.37, *F* (1, 126) =15.49, *p* < 0.001, *η*^2^ = 0.173], and therefore, the pretest operations were valid.

#### Experimental Design and Procedures

In Study 2, the second experiment was designed to examine the mediating effect of anxiety on the main effect. Experiment 2 was a one-way (fluid compensation) and a two-level [with (green product) vs. without (non-green product)] between-subjects factorial design. G*Power 3.1.9.2 (Faul et al., 2007) was used to estimate the planned sample size. Setting a significance level of *α* = 0.05, estimated power of 1-β = 0.80 (requiring more than 0.8, Faul et al., 2007; Taylor and Noseworthy, 2020), and medium effect size *f* = 0.25 (small: *f* = 0.1, medium: *f* = 0.25, large: *f* = 0.4) as the standards to calculate the minimum sample size of the between-factors experiment. This setting refers to previous similar experiments (Huang et al., 2021; Miao et al., 2021), and the result was 128. Considering the possibility of participants not responding seriously in the experiment and the requirement of a greater estimated power, more participants were selected.

Consistent with the operation of Experiment 1, about 150 participants were randomly selected in a comprehensive university in south China and randomly divided into two groups: green product group and non-green product group. At the start of the experiment, participants were asked to listen to soothing music, which was thought to reduce stress and calm the mind and then filled out an anxiety test. Ten measurement items were chosen from the State–Trait Anxiety Inventory (STAI, Spielberger et al., 1970). Five items were applied to measure the trait anxiety (e.g., I feel nervous and restless; I am a steady person: 1 = almost never; 4 = almost always, *α* = 0.715), and five items for state anxiety (e.g., anxious, 1 = not anxious at all; 4 = extremely anxious, *α* = 0.886). This scale was selected in reference to the research of Spielberger and Reheiser (2009). The detailed information is shown in Table 5.

Two groups of participants were asked to comment on a new vacuum cup product and see the pictures of extremely incongruent products to evoke necessary patterns (Meyers-Levy and Tybout, 1989). The only difference between the two groups was the product description, and then, participants were asked to complete the measurement items on the next page.

The participants answered measurement items regarding overall product evaluation, perceived typicality, and incongruity solution. The previous anxiety scale questions on the measurement of state anxiety were then filled out again, and the basic idea of the previous measure of trait anxiety levels was to control participants’ trait anxiety levels in tests for changes in state anxiety (Noseworthy et al., 2014). After that, the participants were asked to fill out the demographic information.

**Table 5:** Measurement scale of anxiety variables.

Variable	Items
State Anxiety	1. I feel secure (r).
	2. I feel anxious.
	3. I feel out of sorts.
	4. I feel stressed and confused.
	5. I feel self-confident (r).
Trait Anxiety	1. I feel nervous and restless.
	2. I wish I could be as happy as others seem to be (r).
	3. I have disturbing thoughts.
	4. I am a steady person (r).
	5. I worry too much over something that really does not matter.

*“R” means the reverse scoring item*.

#### Results and Discussion

Referring to the method of Experiment 1, the invalid questionnaires were deleted based on the reverse question items, and 132 valid participants were retained. The effectiveness rate was 88%, including 67 in the extremely incongruent non-green product group and 65 in the extremely incongruent green product group, among which 64.3% were female, 73.8% were 18–25 years old, 75.7% were bachelor degree or above, and 71.4% were income group of less than 3,000 yuan. And the four-point system of state anxiety and trait anxiety was converted into a seven-point system. The one-way analysis of variance found that fluid compensation had no significant effect on perceived typicality [*F* (1, 130) = 0.015, *p* = 0.902, *η*^2^ < 0.001], indicating that fluid compensation may not have changed the perceived typicality of the product.

As for product evaluation, the main effect of fluid compensation was significant, and specifically, the evaluation of green products was significantly higher than that of non-green products [*M_green product_* = 4.38, *SD* = 0.60, *M_non-green product_* = 4.15, *SD* = 0.63, *F* (1,130) =4.34, *p* = 0.039, *η^2^* = 0.107]. In terms of product incongruity resolution, the effect of fluid compensation was not significant [*F* (1, 130) = 0.015, *p* = 0.904, *η*^2^ < 0.001].

By comparing the levels of state anxiety and trait anxiety of the two groups before the formal experiment, no significant differences were found between the two groups on state anxiety [*F* (1, 126) = 0.023, *p* = 0.877, *η^2^* < 0.001] and trait anxiety [*F* (1, 126) = 0.014, *p* = 0.907, *η^2^ <* 0.001]. However, after the participants scanned through the extremely incongruent vacuum cup and comparing the two groups’ state anxiety, the effect of fluid compensation was significant, and the state anxiety level of green products was significantly lower than that of non-green products [*M_green product_* = 3.86, *SD* = 0.91, *M_non-green product_* = 4.62, *SD* = 0.93, *F* (1, 130) = 14.00, *p* < 0.001, *η*^2^ = 0.314].

In line with the mediating analysis procedure proposed by Zhao et al. (2010) and referring to the Bootstrap method proposed by Preacher and Hayes (2004), the mediating effect test was conducted, Model 4 was selected for the mediating variable test, and the sample size was 5,000, 95% confidence intervals. The results of the mediating test did not contain 0 (*β* = 0.4896, *SE* = 0.0924, *LLCI* = 0.3094, *ULCI* = 0.6702), indicating that the mediating effect of anxiety was significant. Furthermore, after controlling the mediating variable anxiety, the effect of the independent variable fluid compensation on the dependent variable product evaluation was not significant, as the 95% confidence interval contained 0 (*LLCI* = −0.1504, *ULCI* = 0.2263).

Experiment 2 was designed to verify Hypothesis 1 and Hypothesis 2 that the effect of fluid compensation for extremely incongruent products on product evaluation is mediated by the level of anxiety, which was supported by the empirical results. The low degree of perceived typicality of extremely incongruent products awoke anxiety and reduced product evaluation. Fluid compensation for the meaning violation of extremely incongruent products could reduce the level of anxiety and thus improved product evaluation. Although Experiment 2 verified that high-level perceived incongruity awakens high-level anxiety and fluid compensation is conducive to alleviating anxiety levels, whether the effect of fluid compensation for extremely incongruent products has boundaries or not would to be investigated in Experiment 3.

### Study 3

Study 3 was to explore the moderating or boundary role of self-esteem between fluid compensation effects and anxiety for extremely incongruent products. In fact, the boundary role can be seen explored in many similar studies (Liu et al., 2018; Taylor and Noseworthy, 2020). On the one hand, it is to contribute to the enrichment of the theory, and on the other hand, it is to better guide practical activities. In study 3, we assume that self-esteem would affect fluid compensation for the evaluation of extremely incongruent products. The product category chosen for the congruity manipulation was computer mice. A total of 163 college students that were used in Study 1 pretest were randomly divided into three groups to conduct the congruity test of three types of computer mice with obviously different appearances. Participants of each group viewed a picture of one of the mouse on a computer, and they were asked to rate the appearance congruity of the products. Measurement scales of congruity applied the items developed by Noseworthy and Trudel (2011) with the seven-point scale. All participants in the experiment will receive a certain amount of cash reward. According to the data results, the mouse (heterotypic) with appearance typicality below 3 which was the lowest [*M_3_* = 2.40, *F* (1, 161) = 21.42, *p* < 0.001, *η*^2^ = 0.513] was used as the visually extremely incongruent new product for the experiment (Table 6).

**Table 6:** Pretest of computer mice.

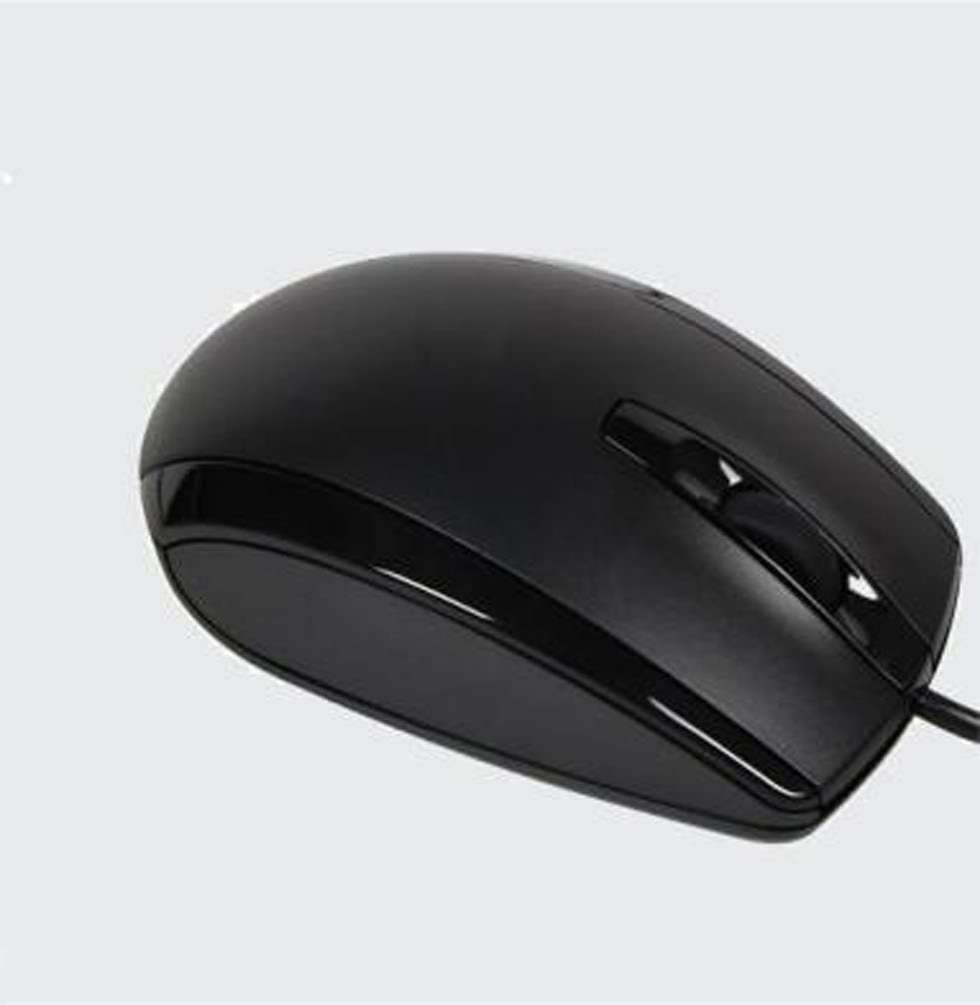 Congruent Product	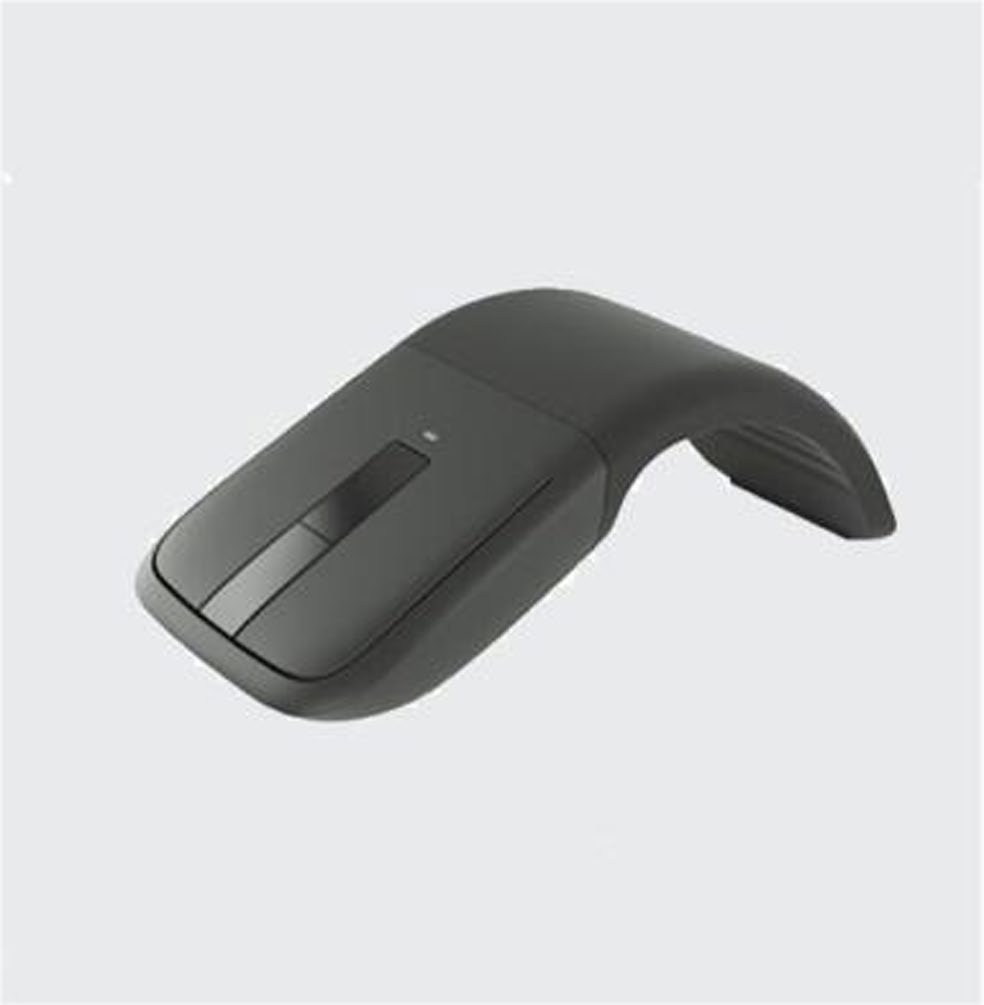 Moderately Incongruent Product	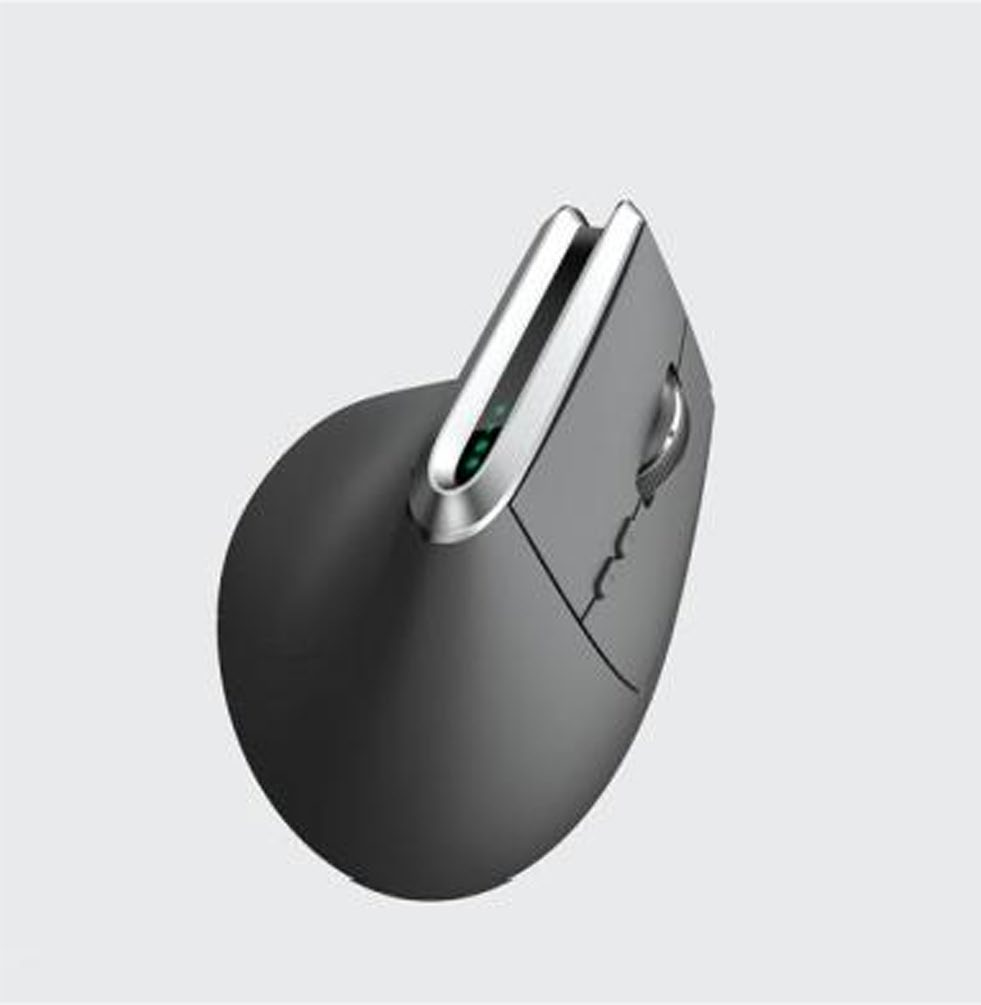 Extremely Incongruent Product
M1 = 5.34	M2 = 3.23	M3 = 2.40
SD = 1.20	SD = 1.12	SD = 1.33

Product description with the same number of words was designed for each type of mouse. The ethnocentric product was described as “the mouse is designed by a designer of a domestic mouse brand, with exquisite craftsmanship,” and the non-ethnocentric product was described as “the mouse is designed by a designer of a foreign mouse brand, with exquisite craftsmanship,” and the rest were in line with the processes of Experiment 1. The product presentation consisted of a product picture (picture of the extremely incongruent product), product name, and brand description; the pictures of the products processed by Photoshop software are of the same size and put above the brand description.

The remaining 128 participants in the pretest of Study 1 were randomly divided into two groups, which were the ethnic product group and the non-ethnic product group, and then, the participants were asked to fill in their own descriptions of which brand the mouse was more likely to be in accord with the product description. This approach can effectively eliminate the influence of brand dominance on the perception of ethnic brands, because the brand that participants first associate with is more prominent and influential in that series of products. To test the participants’ perceptions of the product, the above answers were coded. Ethnic brands were coded as 1, and non-ethnic brands as 0, with reliability being *α* = 0.81. It was found that more participants chose ethnic brands (64% vs. 36%) in the ethnocentric product description and more participants chose non-ethnic brands in the non-ethnocentric product description [70% vs. 30%), *Pearson χ*^2^ (1) = 15.18, *p* < 0. 001], demonstrating that the operation was valid and could be applied to the experiment.

#### Experimental Design and Procedures

In Experiment 3, we designed a 2 (fluid compensation: with (ethnocentric product) vs. without (non-ethnocentric product) × 2 (self-esteem level: high self-esteem vs. low self-esteem) between-subjects factorial design. G*Power 3.1.9.2 (Faul et al., 2007) was used to estimate the planned sample size. Setting a significance level of α = 0.05, estimated power of 1-β = 0.80 (requiring more than 0.8, Faul et al., 2007; Taylor and Noseworthy, 2020), and medium effect size *f* = 0.25 (small: *f* = 0.1, medium: *f* = 0.25, large: *f* = 0.4) as the standards to calculate the minimum sample size of the effects (main effects and interaction effects) of all factors. This setting refers to previous similar experiments (Huang et al., 2021; Miao et al., 2021), and the result was 128. Considering the possibility of participants not responding seriously in the experiment and the requirement of a greater estimated power, more participants were selected.

Following the first two experiments, 150 participants were randomly selected in a comprehensive university in south China and randomly divided into two groups which were the ethnic product group and the non-ethnic product group. All participants were told that they were participating in a consumer survey designed to explore the consumer’s perception of a new computer mouse, and before the experiment, they were asked to listen to soothing music and filled out a state and trait anxiety inventory for which to control the participants’ level of anxiety mainly aroused by the experiment. Then, they saw pictures of the extremely incongruent computer mice with product descriptions on computers and filled in the measurement items on the next page.

The measurement items mainly include overall product evaluation, perceived typicality, incongruent resolution, state anxiety, and individuals’ self-esteem levels. Measurement scale of self-esteem applied the scale of State Self-Esteem proposed by Heatherton and Polivy (1991), from which seven items with a factor loading exceeding 0.50 were selected, including three items measured performance self-esteem (e.g., I feel like I’m not doing very well, 1 = strongly disagree, 4 = strongly agree, *α* = 0.750), two items measured appearance self-esteem (e.g., I feel unattractive, *α* = 0.713), the remaining two measured social self-esteem (e.g., I am worried about what other people think of me, *α* = 0.701). The detailed information is shown in Table 7. The process of the State Self-Esteem scale began with recoding partially reverse-coded entries; then, the coded scores were averaged to form an equally split state self-esteem scale, and those below the median were low self-esteem levels, and those above the median were high self-esteem levels. Then, participants were asked to fill in the demographic information.

**Table 7:** Self-esteem variable measurement scale.

Variable	Primary factor	Items
Self-esteem	Performance self-esteem	I feel confident about my abilities.
		I feel confident that I understand things.
		I feel that I have less scholastic ability right now than others (r).
	Appearance self-esteem	1. I feel satisfied with the way my body looks right now.
		2. I feel unattractive (r).
	Social self-esteem	1. I am worried about whether I am regarded as a success or failure (r).
		2. I am worried about what other people think of me (r).

*“R” means the reverse scoring item*.

#### Results and Discussion

After removing invalid questionnaires, 128 valid participants were retained. The efficiency rate was 85.33%, including 33 in the low self-esteem non-ethnic product group, 30 in the high self-esteem non-ethnic product group, 32 in the low self-esteem ethnic product group, and 33 in the high self-esteem ethnic product group, among which 73.6% were women, 68.8% were people aged 18–25, 78.4% were people with bachelor’s degree or above, and 70.4% were people with an income of less than 3,000 yuan per month. The one-way analysis of variance found that the main effect of fluid compensation was not significant in terms of the product’s perceived typicality [*F* (1, 126) = 0.384, *p* = 0.538, *η*^2^ = 0.030], showing that fluid compensation may not change the perceived typicality of the product and the subsequent experiment can be conducted.

The one-way analysis of variance was selected, with fluid compensation, self-esteem level as the independent variables, and product evaluation as the dependent variable. The results showed that in terms of product evaluation, the main effect of fluid compensation (ethnocentric consumption) was significant [*F* (1, 124) = 13.41, *p* = 0.001, *η*^2^ = 0.294] and the main effect of self-esteem was also significant. The product evaluation made by participants with high-level self-esteem was significantly higher than that with low-level self-esteem [*M_high self-esteem_* = 5.46, *SD* = 0.80, *M_low self-esteem_* = 4.10, *SD* = 1.09, *F* (1, 124) = 37.92, *p* < 0.001, *η*^2^ = 0.532], and the interactive effect of fluid compensation and self-esteem was also significant [*F* (1, 124) = 17.35, *p* < 0.001, *η*^2^ = 0.391]. The simple effect analysis found that the interactive effect of fluid compensation and self-esteem indicated that as for the participants with low-level self-esteem, their evaluation of ethnocentric products was significantly higher than that of non-ethnocentric products [*M_ethnocentric product_* = 5.02, *SD* = 0.21, *M_non-ethnocentric product_* = 3.43, *SD* = 0.18, *F* (1, 124) = 33.04, *p* < 0.001, *η*^2^ = 0.359], and as for the participants with high-level self-esteem, there was no difference between their evaluation of ethnocentric products and non-ethnocentric products [*F* (1, 124) = 2.12, *p* = 0.151, *η*^2^ = 0.002].

**Figure 2:**
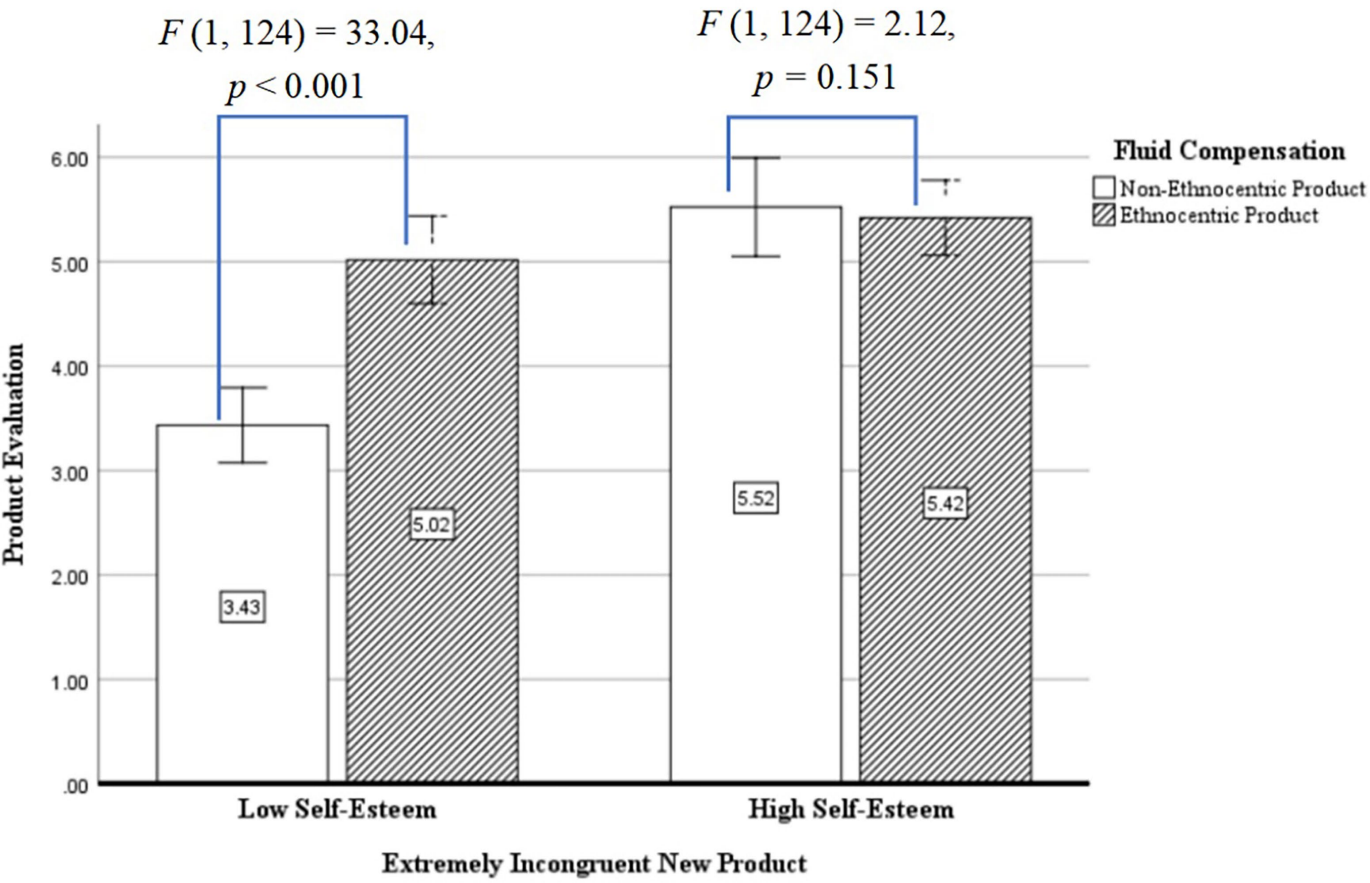
Interactive effects of self-esteem and fluid compensation on product evaluation. Error Bar: 95% confidence interval.

As for the product’s incongruity resolution, the effect of fluid compensation was not significant [*F* (1, 126) = 1.57, *p* = 0.733, *η*^2^ = 0.006]. Additionally, demographic variables such as gender, age, education background, and income had no significant effect on product evaluation. And the effect of fluid compensation was not significant, as shown in Figure 2.

**Figure 3:**
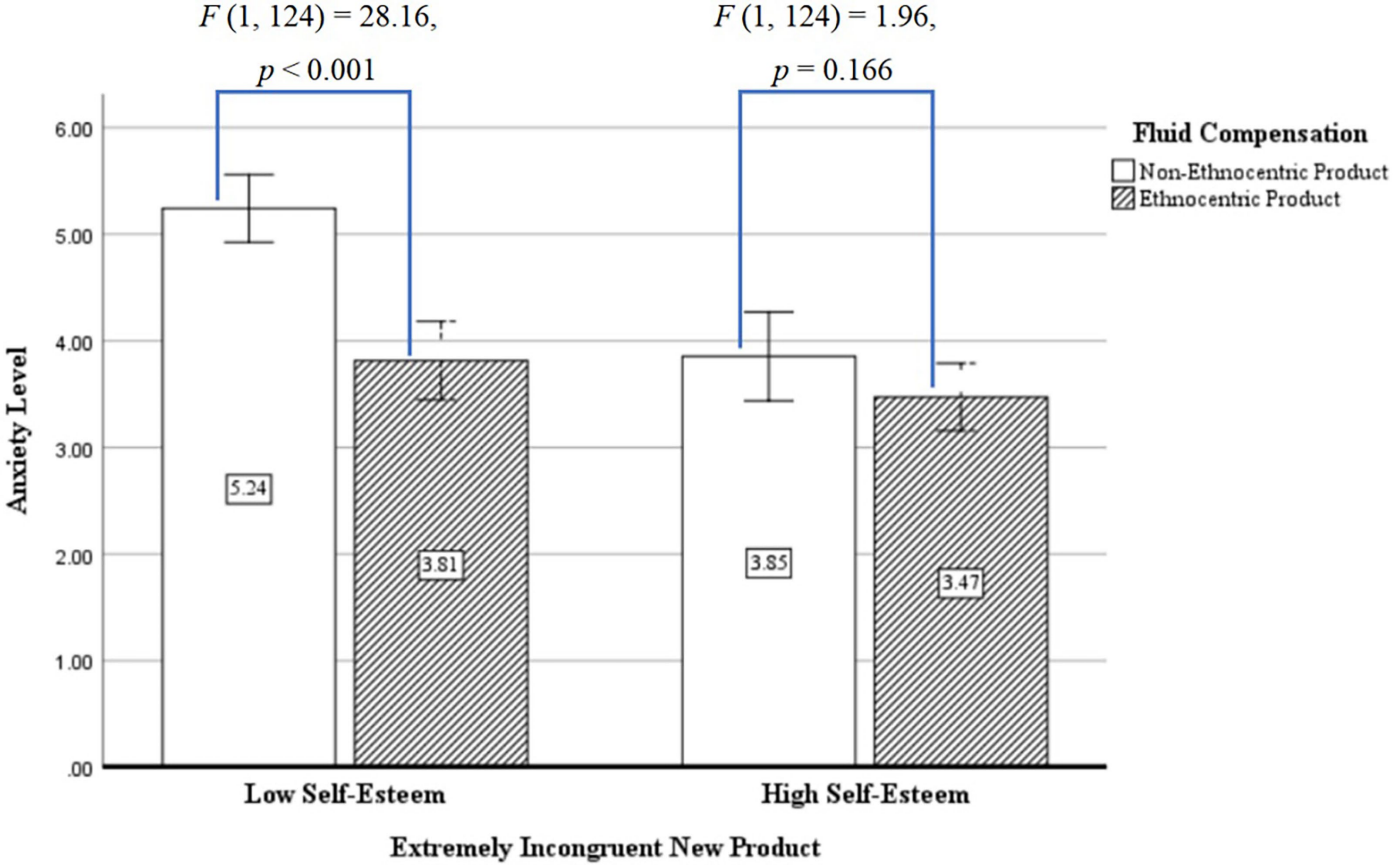
Interactive effects of self-esteem and fluid compensation on anxiety. Error Bar: 95% confidence interval.

No significant difference was found between the four groups on state anxiety (*F* (1, 126) = 0.78, *p* = 0.452, *η^2^* = 0.002) and trait anxiety (*F* (1, 126) = 1.05, *p* = 0.309, *η^2^* = 0.012). The one-way analysis of variance was made, with fluid compensation and self-esteem as the independent variables and state anxiety as the dependent variable. The results showed that in terms of state anxiety, the main effect of fluid compensation (ethnocentric consumption) was significant, for that the level of state anxiety aroused with fluid compensation (ethnocentric product) was significantly lower than the level of state anxiety aroused without fluid compensation (non-ethnocentric product; *M_ethnocentric product_* = 3.62, *SD* = 0.75, *M_non-ethnocentric product_* = 4.73, *SD* = 0.92, *F* (1, 124) = 25.66, *p* < 0.001, *η^2^* = 0.303); the main effect of self-esteem was also significant, the level of anxiety aroused by participants with high self-esteem was significantly lower than that of participants with low self-esteem [*M_high self-esteem_* = 3.61, *SD* = 0.70, *M_low self-esteem_* = 4.64, *SD* = 0.99, *F* (1, 124) = 23.42, *p* < 0.001, *η^2^* = 0.284]; and the interactive effect of fluid compensation and self-esteem was also significant [*F* (1, 124) = 8.60, *p* < 0.005, *η*^2^ = 0.127], and the results of simple effect analysis indicated that in terms of the interactive effect of fluid compensation and self-esteem, the level of anxiety of the participants with low self-esteem with fluid compensation (ethnocentric product) was significantly lower than that without fluid compensation [non-ethnocentric product; *M_ethnocentric product_* = 3.81, *SD* = 0.18, *M_non-ethnocentric product_* = 5.24, *SD* = 0.16, *F* (1, 124) = 28.16, *p* < 0.001, *η*^2^ = 0.323]. However, the level of anxiety of the participants with high self-esteem was not affected with or without fluid compensation [*F* (1, 124) = 1.96, *p* = 0.166, *η*^2^ = 0.032], and thus, the effect of fluid compensation was not significant, and the detailed information is shown in Figure 3.

Additionally, the mediating effect of anxiety on the main effect was retested by using the method of Experiment 2, and the result showed that the 95% confidence interval did not contain 0 (*β* = 0.6028, *SE* = 0.2640, *LLCI* = 0.1612, *ULCI* = 1.1979), indicating that the mediating effect of anxiety was significant, and Experiment 2 was verified once again.

Experiment 3 verified Hypothesis 1 and Hypothesis 2 while testing Hypothesis 3. The effect of fluid compensation for extremely incongruent products on product evaluation is moderated by the level of self-esteem. As for the extremely incongruent product, the fluid compensation of the participants with low self-esteem reduces the individual’s anxiety level and thus improves the product evaluation, whereas the effect of fluid compensation was not significant on the participants with high self-esteem. The reason is that as for extremely incongruent products, the participants with low self-esteem do not think they can solve product incongruity and they need to reduce the anxiety level through fluid compensation to improve the product evaluation, whereas the participants with high self-esteem are confident to solve product incongruity, and the level of anxiety awakened by extremely incongruent new product was low; thus, no matter with or without fluid compensation, the product evaluation is not changed. Theoretically, for those with high self-esteem levels, the presence or absence of fluid compensation does not change the level of anxiety in the face of extremely incongruent products, so product evaluations remain unchanged. For people with low self-esteem levels, the presence or absence of fluid compensation will significantly change the anxiety level and thus the product evaluation. For companies, in the process of marketing new products, differentiated marketing is carried out by analyzing the characteristics of the product’s target customers. Specifically, if the target customer has a high level of self-esteem, the width and depth of product innovation can be increased. And if the target of the product service is mostly low self-esteem, then the emphasis on the sense of meaning related to the product needs to be increased in the product marketing process.

## General Discussion and Implications

Based on the meaning maintenance model, this paper focused on the fluid compensation theory to explore the effect of fluid compensation on the evaluation of extremely incongruent products as well as the intrinsic mechanism and boundary conditions under which the effect occurs. Three experiments were conducted to verify the hypotheses. Experiment 1 designed a 2 (product congruity: congruent product vs. extremely incongruent product) × 2 [fluid compensation: with (dominant brand) vs. without (non-dominant brand)] intergroup experiment to explore whether fluid compensation changes product evaluation of extremely incongruent products; Experiment 2 designed an one-way [fluid compensation: with (green product) vs. without (non-green product)] intergroup experiment to explore the mediating mechanism for fluid compensation to change the evaluation of extremely incongruent products; Experiment 3 designed a 2 [fluid compensation: with (ethnocentric product) vs. without (non-ethnocentric product)] × 2 (self-esteem: high level vs. low level) intergroup experiment to explore the moderating effect of the individual’s self-esteem level on the state anxiety aroused by extremely incongruent products.

The research results showed that compared with congruent products, extremely incongruent products will bring lower product evaluation, and the product evaluation will be improved through fluid compensation approaches such as dominant brand preference, green consumption concept, and ethnocentric view of consumption because the aversion caused by the meaning violation is compensated. The aversion caused by meaning violation is mainly manifested as anxiety (state anxiety) aroused by schema incongruity, and fluid compensation reduces the anxiety level and thus improves product evaluation. In addition, the effect of fluid compensation varies from person to person, specifically, as for individuals with low self-esteem, the fluid compensation of extremely incongruent products will reduce the individual’s state anxiety level and thus improves product evaluation; nevertheless, as for individuals with high self-esteem, the effect of fluid compensation is not significant. The approaches of fluid compensation in Experiment 1, Experiment 2, and Experiment 3, including dominant brand preference, green consumption concept, and ethnocentric view of consumption, are proved to have a positive effect on product evaluation of extremely incongruent products.

### Theoretical Implications

First, this paper explores the strategies to improve the evaluation of extremely incongruent products from a new theoretical perspective. It explores the effect of the indirect strategy of the meaning maintenance model, fluid compensation, on the evaluation of extremely incongruent products based on assimilation and compliance of existing studies on schema congruity. Much of the academic literature has studied the theory before, and what previous studies mainly focused on is that product incongruity in a field will affirm the other products in another field to maintain the existing meaning to improve the product evaluation (Taylor and Noseworthy, 2020). However, limited research resolved incongruity in a belief structure that can be satisfied by affirming another belief structure. In this study, the product range of fluid compensation is narrowed, with its focus on the meaning maintenance of the product itself, for instance, consumers affirm the meaning brought by the product consumption (dominant brand preference, green consumption, ethnocentric view of consumption, and other beliefs), instead of changing or updating product models, and there is no need to borrow other products to maintain the meaning. This study is consistent with the findings of previous research by Liu et al. (2018) on extremely incongruent products, holding that the negative evaluation of extremely incongruent products can be improved through relevant strategies.

Second, this paper explores the intrinsic mechanism of fluid compensation for extremely incongruent products, namely extreme incongruity awakens anxiety and fluid compensation reduces the anxiety level to enhance the product evaluation. This is also consistent with previous research findings that uncertainty will arouse anxiety (Noseworthy et al., 2014). Previous studies have also shown that when the source of anxiety is unclear, it can be controlled through a wider range of paths (Raghunathan and Pham, 1999). Our findings also indicate that anxiety awakened by extremely incongruent products is unconscious and it can be alleviated by fluid compensation.

Third, this paper also explores the moderating effect of the self-esteem on state anxiety aroused by incongruent products. Limited research has focused on the interactive effects of self-esteem and anxiety on incongruent products. We come to the conclusion that whether the personal sense of control is threatened or not will be determined by the individual’s self-esteem level, and those with high self-esteem tend to have a higher sense of self-value and higher self-evaluation, which are conducive to dealing with threats. The extremely incongruent product in this study means that the schematic meaning of the product is threatened and an individual’s self-esteem may also affect the fluid compensation of the incongruent product, and the role of self-esteem in the extent of threat on meaning is confirmed in this study.

### Managerial Implications

The effect of fluid compensation for extremely incongruent products studied in this paper may play a role in selling new products. If the product is a new visual product, it can affirm the positive role related to the product as much as possible to make consumers believe that the purchase of the product is meaningful rather than invalid consumption. Three approaches of fluid compensation have been explored and verified in this study including dominant brand preference, green consumption concept, and ethnocentric view of consumption, which can be directly used in product marketing.

The reason for enterprises to adopt meaningful ways to improve the evaluation of extremely incongruent products is mainly to ease consumers’ psychological anxiety when seeing incongruent products (Noseworthy et al., 2014). Since extremely incongruent products are likely to cause anxiety, enterprises can take relevant measures to reduce or transfer the anxiety, for instance, displaying extremely comfortable pictures in the sales scene, playing relaxing music, or diffusing perfumes to soothe the nervous atmosphere, etc. These ways may reduce consumers’ anxious perception of extremely incongruent products. Additionally, enterprises can also attribute anxiety to different reasons to form a better product evaluation. Enterprises also need to analyze the features of targeted consumers and determine the degree of product innovation that can be accepted by the market through the portrait of consumers. The comprehensive application of these factors will bring a higher acceptance rate and more profits for enterprises in the process of product innovation.

It is worth mentioning that the introduction of extremely incongruent products will have great implications for public policymakers, primarily in those extremely incongruent products that promote consumers’ pursuit of the sense of meaning, for example, they will buy more green products or identify with domestic products so that consumption is promoted, which is conducive to environmental protection and people’s cultural identity. Thus, the strategic use of extremely incongruent products may exert positive impacts on society indirectly.

### Limitations and Future Direction

Although the research results of this paper are basically consistent with expectations, there are still weaknesses and deficiencies. In terms of research content, this paper discusses the effect of indirect strategy (i.e., fluid compensation) under the meaning maintenance model on the evaluation of incongruent products, while another strategy, extraction, has not been studied yet. It is not known whether these strategies are effective in dealing with incongruent products. The relative efficacy of these strategies under the meaning maintenance model can be further explored in the future. Besides, future research could investigate other ways or fields of compensation for new findings. For example, consumers can compensate for the anxiety caused by meaning violation by affirming their self-worth (Mandel et al., 2017). In addition, future research could also explore how unrelated compensatory behaviors are required for a belief system to be activated. For example, whether a dominant brand in a category obtains beneficial effects from introducing extremely incongruent products in its own category. The findings of the present study shed light on the boundaries of fluid compensation while providing unique managerial insights to better understand how enterprises directly benefit from extreme innovation in their product categories. It is better to explore other possible compensation behaviors, and such exploration contributes to providing more favorable new product marketing strategies.

## Data Availability Statement

The raw data supporting the conclusions of this article will be made available by the authors, without undue reservation.

## Ethics Statement

The studies involving human participants were reviewed and approved by East China Jiaotong University. The patients/participants provided their written informed consent to participate in this study. Written informed consent was obtained from the individual(s) for the publication of any potentially identifiable images or data included in this article.

## Author Contributions

XL contributed to the empirical work, to the analysis of the results, and to the writing of the first draft. YC, XS, and XZ supported the total work of XL. XL contributed to overall quality and supervised the part of literature organization and empirical work. All authors discussed the results and commented on the manuscript.

## Funding

This study is supported by the funding of National Natural Science Foundation of China (No. 71962009), Key Project of Education Science in Jiangxi Province (No. 20ZD028), Management Science Project of Jiangxi Science and Technology Department (No. 2021BBA10007), and Jiangxi Province Colleges Humanities and Social Science Project (No. GL21130).

## Conflict of Interest

The authors declare that the research was conducted in the absence of any commercial or financial relationships that could be construed as a potential conflict of interest.

## Publisher’s Note

All claims expressed in this article are solely those of the authors and do not necessarily represent those of their affiliated organizations, or those of the publisher, the editors and the reviewers. Any product that may be evaluated in this article, or claim that may be made by its manufacturer, is not guaranteed or endorsed by the publisher.

## References

[ref1] Alexander D. L. Lynch J. G. Jr. Qing W. (2008). As time goes by: do cold feet follow warm intentions for really new versus incrementally new products? J. Mark. Res. 45, 307–319. doi: 10.1509/jmkr.45.3.307

[ref2] Ali S. A. A. Diab S. S. E. M. Elmahallawy E. K. (2021). Exploring the psychological stress, anxiety factors, and coping mechanisms of critical care unit nurses during the COVID-19 outbreak in Saudi Arabia. Front. Public Health 9:767517. doi: 10.3389/fpubh.2021.767517, PMID: 34900913 PMC8661107

[ref3] Awan U. Arnold M. G. Gölgeci I. (2021). Enhancing green product and process innovation: towards an integrative framework of knowledge acquisition and environmental investment. Bus. Strateg. Environ. 30, 1283–1295. doi: 10.1002/bse.2684

[ref4] Bagga C. K. Noseworthy T. J. Dawar N. (2016). Asymmetric consequences of radical innovations on category representations of competing brands. J. Consum. Psychol. 26, 29–39. doi: 10.1016/j.jcps.2015.04.005

[ref5] Berger U. Keshet H. Gilboa-Schechtman E. (2017). Self-evaluations in social anxiety: the combined role of explicit and implicit social-rank. Personal. Individ. Differ. 104, 368–373. doi: 10.1016/j.paid.2016.08.023

[ref6] Brailovskaia J. Margraf J. (2021). The relationship between burden caused by coronavirus (covid-19), addictive social media use, sense of control and anxiety. Comput. Hum. Behav. 119:106720. doi: 10.1016/j.chb.2021.106720, PMID: 33785982 PMC7994028

[ref7] Bruner J. S. (1990). Acts of Meaning. Cambridge, MA: Harvard University Press.

[ref8] Burmeister E. Aitken L. M. (2012). Sample size: how many is enough? Aust. Crit. Care 25, 271–274. doi: 10.1016/j.aucc.2012.07.002 22835279

[ref9] Campbell M. C. Goodstein R. C. (2001). The moderating effect of perceived risk on consumers’ evaluations of product incongruity: preference for the norm. J. Consum. Res. 28, 439–449. doi: 10.1086/323731

[ref10] Clemente S. Dolansky E. Mantonakis A. White K. (2013). The effects of perceived product-extrinsic cue incongruity on consumption experiences: the case of celebrity sponsorship. Mark. Lett. 25, 373–384. doi: 10.1007/s11002-013-9257-y

[ref11] De Guimarães J. C. F. Severo A. E. Jabbourb C. J. C. Jabbourb A. B. L. S. Rosa A. F. P. (2021). The journey towards sustainable product development: why are some manufacturing companies better than others at product innovation? Technovation 103:102239. doi: 10.1016/j.technovation.2021.102239

[ref12] Dhandra T. K. (2020). Does self-esteem matter? A framework depicting role of self-esteem between dispositional mindfulness and impulsive buying. J. Retail. Consum. Serv. 55:102135. doi: 10.1016/j.jretconser.2020.102135

[ref13] Dumas T. M. Maxwell-Smith M. A. Tremblay P. F. Litt D. M. Ellis W. (2020). Gaining likes, but at what cost? Longitudinal relations between young adults' deceptive like-seeking on instagram, peer belonging and self-esteem. Comput. Hum. Behav. 112:106467. doi: 10.1016/j.chb.2020.106467

[ref14] Duong P. N. Voordecker W. Huybrechts J. Lambrechts F. (2022). On external knowledge sources and innovation performance: family versus non-family firms. Technovation 114:102448. doi: 10.1016/j.technovation.2021.102448

[ref15] Faul F. Erdfelder E. Lang A. G. Buchner A. (2007). G* power 3: a flexible statistical power analysis program for the social, behavioral, and biomedical sciences. Behav. Res. Methods 39, 175–191. doi: 10.3758/BF03193146, PMID: 17695343

[ref16] Forehand M. Reed A. Saint Clair J. K. (2021). Identity interplay: the importance and challenges of consumer research on multiple identities. Consum. Psychol. Rev. 4, 100–120. doi: 10.1002/arcp.1066

[ref17] Gourville J. T. (2006). Eager sellers and stony buyers: understanding the psychology of new-product adoption. Harv. Bus. Rev. 84, 98–106, 145. PMID: . 16770897

[ref18] Griskevicius V. Tybur J. M. Van Den Bergh B. (2010). Going green to be seen: status, reputation, and conspicuous conservation. J. Pers. Soc. Psychol. 98, 392–404. doi: 10.1037/a0017346, PMID: 20175620

[ref19] Gruda D. Hasan S. (2019). Feeling anxious? Perceiving anxiety in tweets using machine learning. Comput. Hum. Behav. 98, 245–255. doi: 10.1016/j.chb.2019.04.020

[ref20] Heatherton T. F. Polivy J. (1991). Development and validation of a scale for measuring state self-esteem. J. Pers. Soc. Psychol. 60, 895–910. doi: 10.1037/0022-3514.60.6.895

[ref21] Holte A. J. Ferraro F. R. (2020). Anxious, bored, and (maybe) missing out: evaluation of anxiety attachment, boredom proneness, and fear of missing out (fomo). Comput. Hum. Behav. 112:106465. doi: 10.1016/j.chb.2020.106465

[ref22] Hong W. Liu R. D. Oei T. P. (2019). The mediating and moderating roles of social anxiety and relatedness need satisfaction on the relationship between shyness and problematic mobile phone use among adolescents. Comput. Hum. Behav. 93, 301–308. doi: 10.1016/j.chb.2018.12.020

[ref23] Huang J. Wan X. (2019). The color–flavor incongruency effect in product evaluation and brand perception. J. Consum. Behav. 18, 484–495. doi: 10.1002/cb.1787

[ref24] Huang Y. Zhang B. Fan X. Huang J. (2021). Can negative emotion of task-irrelevant working memory representation affect its attentional capture? A study of eye movements. Acta Psychol. Sin. 53, 26–37. doi: 10.3724/SP.J.1041.2021.00026

[ref25] Jabeen G. Ahmad M. Zhang Q. (2021). Perceived critical factors affecting consumers’ intention to purchase renewable generation technologies: rural-urban heterogeneity. Energy 218:119494. doi: 10.1016/j.energy.2020.119494

[ref26] Jhang J. H. Grant S. J. Campbell M. C. (2012). Get it? Got it. Good! Enhancing new product acceptance by facilitating resolution of extreme incongruity. J. Mark. Res. 49, 247–259. doi: 10.1509/jmr.10.0428

[ref27] Kamalou S. Shaughnessy K. Moscovitch D. A. (2019). Social anxiety in the digital age: the measurement and sequelae of online safety-seeking-sciencedirect. Comput. Hum. Behav. 90, 10–17. doi: 10.1016/j.chb.2018.08.023

[ref28] Kim E. Koh E. (2018). Avoidant attachment and smartphone addiction in college students: the mediating effects of anxiety and self-esteem. Comput. Hum. Behav. 84, 264–271. doi: 10.1016/j.chb.2018.02.037

[ref29] Kolling C. Ribeiro J. L. D. de Medeiros J. F. (2022). Performance of the cosmetics industry from the perspective of corporate social responsibility and design for sustainability. Sustainable Prod. Consumption 30, 171–185. doi: 10.1016/j.spc.2021.12.002

[ref30] Lee M. Septianto F. Frethey-Bentham C. Gao E. (2020). Condoms and bananas: shock advertising explained through congruence theory. J. Retail. Consum. Serv. 57:102228. doi: 10.1016/j.jretconser.2020.102228

[ref31] Li J. Fang M. Wang W. Sun G. Cheng Z. (2018b). The influence of grit on life satisfaction: self-esteem as a mediator. Psychol. Belgica 58, 51–66. doi: 10.5334/pb.400, PMID: 30479807 PMC6194520

[ref32] Li D. Jin H. Zheng J. (2018a). The impact of product assortment on the evaluation of extremely incongruent new products. Manag. Rev. 30, 97–109. doi: 10.14120/j.cnki.cn11-5057/f.2018.09.009

[ref33] Li X. Katsumata S. (2020). The impact of multidimensional country distances on consumption of specialty products: a case study of inbound tourists to Japan. J. Vacat. Mark. 26, 18–32. doi: 10.1177/1356766719842280

[ref34] Li X. Wirawan D. Ye Q. Peng L. Zhou J. (2021). How does shopping duration evolve and influence buying behavior? The role of marketing and shopping environment. J. Retail. Consum. Serv. 62:102607. doi: 10.1016/j.jretconser.2021.102607

[ref35] Liu W. Lei L. Li Z. Su Y. Huang X. (2018). Touch or not touch? Prior touch facilitates consumers’ adoption of new products. Acta Psychol. Sin. 50, 782–792. doi: 10.3724/SP.J.1041.2018.00782

[ref36] Major B. Townsend S. S. (2012). Meaning making in response to unfairness. Psychol. Inq. 23, 361–366. doi: 10.1080/1047840X.2012.722785

[ref37] Mandel N. Lisjak M. Wang Q. (2021). Compensatory routes to object attachment. Curr. Opin. Psychol. 39, 55–59. doi: 10.1016/j.copsyc.2020.07.026, PMID: 32823243

[ref38] Mandel N. Rucker D. D. Levav J. Galinsky A. D. (2017). The compensatory consumer behavior model: how self-discrepancies drive consumer behavior. J. Consum. Psychol. 27, 133–146. doi: 10.1016/j.jcps.2016.05.003

[ref39] Meyers-Levy J. Tybout A. M. (1989). Schema congruity as a basis for product evaluation. J. Consum. Res. 16, 39–54. doi: 10.1086/209192

[ref40] Miao X. Sun X. Kuang Y. Wang Z. (2021). Co-experiencing the same negative emotional events promotes cooperation. Acta Psychol. Sin. 53, 81–94. doi: 10.3724/SP.J.1041.2021.00081

[ref41] Najafi-Tavani S. Najafi-Tavani Z. Naudé P. Oghazi P. Zeynaloo E. (2018). How collaborative innovation networks affect new product performance: product innovation capability, process innovation capability, and absorptive capacity. Ind. Mark. Manag. 73, 193–205. doi: 10.1016/j.indmarman.2018.02.009

[ref42] Narang R. (2016). Understanding purchase intention towards Chinese products: role of ethnocentrism, animosity, status and self-esteem. J. Retail. Consum. Serv. 32, 253–261. doi: 10.1016/j.jretconser.2016.05.010

[ref43] Noseworthy T. J. Cotte J. Lee S. H. (2011). The effects of ad context and gender on the identification of visually incongruent products. J. Consum. Res. 38, 358–375. doi: 10.1086/658472

[ref44] Noseworthy T. J. Goode M. R. (2011). Contrasting rule-based and similarity-based category learning: The effects of mood and prior knowledge on ambiguous categorization. J. Consum. Psychol. 21, 362–371. doi: 10.1016/j.jcps.2011.03.003

[ref45] Noseworthy T. J. Muro F. D. Murray K. B. (2014). The role of arousal in congruity based product evaluation. J. Consum. Res. 41, 1108–1126. doi: 10.1086/678301

[ref46] Noseworthy T. J. Trudel R. (2011). Looks interesting, but what does it do? Evaluation of incongruent product form depends on positioning. J. Mark. Res. 48, 1008–1019. doi: 10.1509/jmr.10.0384

[ref47] Pleyers G. (2021). Shape congruence in product design: impacts on automatically activated attitudes. J. Retail. Consum. Serv. 61:101935. doi: 10.1016/j.jretconser.2019.101935

[ref48] Preacher K. J. Hayes A. F. (2004). SPSS and SAS procedures for estimating indirect effects in simple mediation models. Behav. Res. Methods Instrum. Comput. 36, 717–731. doi: 10.3758/BF03206553, PMID: 15641418

[ref49] Proulx T. Heine S. J. (2008). The case of the transmogrifying studyer: affirmation of a moral schema following implicit change detection. Psychol. Sci. 19, 1294–1300. doi: 10.1111/j.1467-9280.2008.02238.x, PMID: 19121140

[ref50] Proulx T. Heine S. J. (2010). The frog in kierkegaard’s beer: finding meaning in the threat-compensation literature. Social Personality Psychol. Compass 4, 889–905. doi: 10.1111/j.1751-9004.2010.00304.x

[ref51] Proulx T. Inzlicht M. (2012). The five “A” s of meaning maintenance: finding meaning in the theories of sense-making. Psychol. Inq. 23, 317–335. doi: 10.1080/1047840X.2012.702372

[ref52] Pyszczynski T. Greenberg J. Solomon S. Arndt J. Schimel J. (2004). Why do people need self-esteem? A theoretical and empirical review. Psychol. Bull. 130, 435–468. doi: 10.1037/0033-2909.130.3.435, PMID: 15122930

[ref53] Qiu L. Jie X. Wang Y. Zhao M. (2020). Green product innovation, green dynamic capability, and competitive advantage: evidence from chinese manufacturing enterprises. Corp. Soc. Responsib. Environ. Manag. 27, 146–165. doi: 10.1002/csr.1780

[ref54] Raghunathan R. Pham M. T. (1999). All negative moods are not equal: motivational influences of anxiety and sadness on decision making. Organ. Behav. Hum. Decis. Process. 79, 56–77. doi: 10.1006/obhd.1999.2838 10388609

[ref55] Raghunathan R. Pham M. T. Corfman K. P. (2006). Informational properties of anxiety and sadness, and displaced coping. J. Consum. Res. 32, 596–601. doi: 10.1086/500491

[ref56] Randles D. Proulx T. Heine S. J. (2011). Turn-frogs and careful sweaters: non-conscious perception of incongruous word pairings provokes fluid compensation. J. Exp. Social Psychol. 47, 246–249. doi: 10.1016/j.jesp.2010.07.020

[ref57] Sadiq M. Adil M. Paul J. (2021). An innovation resistance theory perspective on purchase of eco-friendly cosmetics. J. Retail. Consum. Serv. 59:102369. doi: 10.1016/j.jretconser.2020.102369

[ref58] Simpson C. A. Diaz-Arteche C. Eliby D. Schwartz O. S. Simmons J. G. Cowan C. S. (2021). The gut microbiota in anxiety and depression–A systematic review. Clin. Psychol. Rev. 83:101943. doi: 10.1016/j.cpr.2020.101943, PMID: 33271426

[ref59] Sowislo J. F. Orth U. (2013). Does low self-esteem predict depression and anxiety? A meta-analysis of longitudinal studies. Psychol. Bull. 139, 213–240. doi: 10.1037/a0028931, PMID: 22730921

[ref60] Spielberger C. D. Gorsuch R. L. Lushene R. D. (1970). Stai: Manual for the State-Trait Anxiety Inventory. Palo Alto, CA: Consulting Psychologists Press.

[ref61] Spielberger C. D. Reheiser E. C. (2009). Assessment of emotions: anxiety, anger, depression, and curiosity. Appl. Psychol. Health Well Being 1, 271–302. doi: 10.1111/j.1758-0854.2009.01017.x

[ref62] Stojčić N. (2021). Social and private outcomes of green innovation incentives in European advancing economies. Technovation 104:102270. doi: 10.1016/j.technovation.2021.102270

[ref63] Sun G. Wang W. Cheng Z. Li J. Chen J. (2017). The intermediate linkage between materialism and luxury consumption: evidence from the emerging market of China. Soc. Indic. Res. 132, 475–487. doi: 10.1007/s11205-016-1273-x

[ref64] Taylor N. Noseworthy T. J. (2020). Compensating for innovation: extreme product incongruity encourages consumers to affirm unrelated consumption schemas. J. Consum. Psychol. 30, 77–95. doi: 10.1002/jcpy.1127

[ref65] Tesser A. (2000). On the confluence of self-esteem maintenance mechanisms. Personal. Soc. Psychol. Rev. 4, 290–299. doi: 10.1207/S15327957PSPR0404_1

[ref66] Tibber M. S. Zhao J. Butler S. (2020). The association between self-esteem and dimensions and classes of cross-platform social media use in a sample of emerging adults–evidence from regression and latent class analyses. Comput. Hum. Behav. 109:106371. doi: 10.1016/j.chb.2020.106371

[ref67] Waheed A. Zhang Q. Rashid Y. Tahir M. S. Zafar M. W. (2020). Impact of green manufacturing on consumer ecological behavior: stakeholder engagement through green production and innovation. Sustain. Dev. 28, 1395–1403. doi: 10.1002/sd.2093

[ref68] Wang W. Ma T. Li J. Zhang M. (2020). The pauper wears Prada? How debt stress promotes luxury consumption. J. Retail. Consum. Serv. 56:102144. doi: 10.1016/j.jretconser.2020.102144

[ref69] Wang W. Yi Y. Li J. Sun G. Zhang M. (2022). Lighting up the dark: how the scarcity of childhood resources leads to preferences for bright stimuli. J. Bus. Res. 139, 1155–1164. doi: 10.1016/j.jbusres.2021.10.058

[ref70] Xie X. Huo J. Zou H. (2019). Green process innovation, green product innovation, and corporate financial performance: a content analysis method. J. Bus. Res. 101, 697–706. doi: 10.1016/j.jbusres.2019.01.010

[ref71] Xie X. Tang X. Rapp H. Tong D. Wang P. (2020). Does forgiveness alleviate depression after being phubbed for emerging adults? The mediating role of self-esteem. Comput. Hum. Behav. 109:106362. doi: 10.1016/j.chb.2020.106362

[ref72] Zhao X. Lynch J. G. Chen Q. (2010). Reconsidering baron and kenny: myths and truths about mediation analysis. J. Consum. Res. 37, 197–206. doi: 10.1086/651257

[ref73] Zhou Z. Zheng F. Lin J. Zhou N. (2021). The interplay among green brand knowledge, expected eudaimonic well-being and environmental consciousness on green brand purchase intention. Corp. Soc. Responsib. Environ. Manag. 28, 630–639. doi: 10.1002/csr.2075

[ref74] Zhu Z. Li X. Liu F. (2020). How visual novelty affects consumer purchase intention: the moderating effects of self-construal and product type. Acta Psychol. Sin. 52:1352. doi: 10.3724/SP.J.1041.2020.01352

[ref75] Zohreh F. Ahmad A. E. Fazlollah G. Firoozeh M. Ali A. E. (2007). Relationship between self-concept, self-esteem, anxiety, depression and academic achievement in adolescents. J. Appl. Sci. 7, 995–1000. doi: 10.3923/jas.2007.995.1000

